# Modulation of naïve mesenchymal stromal cells by extracellular vesicles derived from insulin-producing cells: an in vitro study

**DOI:** 10.1038/s41598-024-68104-4

**Published:** 2024-08-01

**Authors:** Mahmoud M. Gabr, Sawsan M. El-Halawani, Ayman F. Refaie, Sherry M. Khater, Amani M. Ismail, Mary S. Karras, Raghda W. Magar, Shorouk El Sayed, Malgorzata Kloc, Ahmed Uosef, Omaima M. Sabek, Mohamed A. Ghoneim

**Affiliations:** 1 Biotechnology Department, Urology and Nephrology Center, Mansoura, Egypt; 2 Nephrology Department, Urology and Nephrology Center, Mansoura, Egypt; 3 Pathology Department, Urology and Nephrology Center, Mansoura, Egypt; 4 Immunology Department, Urology and Nephrology Center, Mansoura, Egypt; 5 Microbiology Department, Faculty of Veterinary Medicine, Zagazig University, Zagazig, Egypt; 6 The Houston Methodist Research Institute, Houston, TX USA; 7 Department of Surgery, Houston Methodist Hospital, Houston, TX USA; 8 Department of Genetics, MD Anderson Cancer Center, University of Texas, Houston, TX USA; 9 Urology Department, Urology and Nephrology Center, Mansoura, Egypt

**Keywords:** MSCs, Differentiation, Insulin-producing cells, Extracellular vesicles, Exosomes, Diabetes, Stem cells, Endocrinology

## Abstract

This study was to determine whether extracellular vesicles (EVs) derived from insulin-producing cells (IPCs) can modulate naïve mesenchymal stromal cells (MSCs) to become insulin-secreting. MSCs were isolated from human adipose tissue. The cells were then differentiated to generate IPCs by achemical-based induction protocol. EVs were retrieved from the conditioned media of undifferentiated (naïve) MSCs (uneducated EVs) and from that of MSC-derived IPCs (educated EVs) by sequential ultracentrifugation. The obtained EVs were co-cultured with naïve MSCs.The cocultured cells were evaluated by immunofluorescence, flow cytometry, C-peptide nanogold silver-enhanced immunostaining, relative gene expression and their response to a glucose challenge.Immunostaining for naïve MSCs cocultured with educated EVs was positive for insulin, C-peptide, and GAD65. By flow cytometry, the median percentages of insulin-andC-peptide-positive cells were 16.1% and 14.2% respectively. C-peptide nanogoldimmunostaining providedevidence for the intrinsic synthesis of C-peptide. These cells released increasing amounts of insulin and C-peptide in response to increasing glucose concentrations. Gene expression of relevant pancreatic endocrine genes, except for insulin, was modest. In contrast, the results of naïve MSCs co-cultured with uneducated exosomes were negative for insulin, C-peptide, and GAD65. These findings suggest that this approach may overcome the limitations of cell therapy.

## Introduction

Diabetes mellitus (DM) is a metabolic disorder that is a major health concern. Globally,more than 400 million people suffered from DM in 2014 compared to 108 million in 1980^1^. If this trend continues, the number of patients with diabetes is projected to increase to more than 600 million by 2045^2^. DM results from deficient insulin production as in type1 DM (T1DM) or an inability to utilize this hormone as occurs in type 2 DM (T2DM). The pathogenesis of T1DM involves the autoimmune-mediated destruction of insulin-producing β-cells in the pancreatic islets. Administration of exogenous insulin is the main treatment for T1DM patients. Maintenance of appropriate glycemic control can be achieved with insulin therapy for life. However, inaccurate insulin delivery results in lack of glycemic control and/or episodes of hypoglycemia. Furthermore, insulin therapy fails to prevent microvascular complications in many individuals. Transplantation of pancreatic islets or an intact pancreas is an ideal alternative to lifelong treatment with insulin^3^. However, the shortage of cadaveric organs and the need for lifelong immunosuppression are limiting factors. T2DM accounts for the majority of cases of diabetes. This disease can be initially treated by dietary modification and oral medications. Eventually 27% of patientswith diabetes become insulin-dependent. Of these patients, less than half achieve the recommended glycosylated hemoglobin levels (HbA1c), since exogenous insulin cannot provide the tight glycemic control exerted by pancreatic-derived insulin^4^.

Progress in the field of regenerative therapies provides the potential for the generation of insulin-producing cells (IPCs) from a variety of stem cells. To this end, the use of embryonic, neonatal, induced pluripotent and mesenchymal/stromal cells (MSCs) has been reported. MSCs are the most studied and commonly used cells in the field of regenerative medicine for experimental and/or clinical applications. Initially,it was believed that in vivo administration of MSCs would repair damaged tissue viaengraftment and subsequent differentiation. However, many studies have demonstrated that despite of their therapeutic benefits, MSCs are not easily engrafted into target tissue and most of the transplanted cells become trapped in the lungs and eventually die or are destroyed^5^. It was demonstrated that culture medium conditioned by MSCs produced therapeutic effects similar to those of the parent cells in rodent models of acute myocardial infarction^6^ and lung injury^7^. As a result, the paradigm of MSC-mediated function has shifted from cell engraftment to secretome-based signaling. In 2009, Bruno and associates demonstrated that MSCs secrete microvesicles that protect against acute tubular injury^8^. One year later, Lai et al. showed that MSCs released a specific class of vesicles that reduced infarct size in a mouse model^9^.

EventuallyIt is now known that MSCs exert their therapeutic effects by the release of various membrane-surrounded vesicles collectively referred to as extracellular vesicles (EVs) into the extracellular milieu^10^. EVs are lipid bilayer vesicles and have 3 main types:exosomes, microvesicles and apoptotic bodiesdepending on their size and biogenesis^11^. These vesicles are characterized by their morphology using transmission electron microscopy(TEM), their size by dynamic light scanning and their protein content by Western blotting^12^. The first and best- studied category is exosomes. These exosomes are derived by invagination of the endosomal membrane to form multivesicular bodies (MVBs) which enclose numerous intraluminal vesicles. MVBs are released as exosomes upon fusion with the plasma membrane and have a size of 50–150 nm depending on the method used for analysis. The second major type of vesicles is microvesicles (MVs) which are larger than exosomes and have a size of 100–1000 nm. EVs are released by direct outward budding and fission of the plasma membrane. The third class of EVs are apoptotic bodies formed from cells that undergo programmed cell death and become fragmented. These vesicles are larger ranging from 500 nm to several micronsin size^13^.

EVs carry a cargo of proteins, lipids, and different types of RNA that can be transferred from donor cells to recipient cells^14,15^.EV-mediated cell-to-cell communication involves 3 main mechanisms^16,17^. Proteins within the EV membrane can serve as ligands for receptors on the surface of recipient cells. The ligand-receptor interactions activate signaling pathways. Fusion of the EV-membrane with the recipient cell membrane results in the releaseof EV-contents and theinitiation of different downstream pathways. EVs can also be internalized by endocytosis but this process may lead to the degradation of macromolecules. The delivery of EV cargo into the recipient cells results in epigenetic changes and the modulation of cell function^18–23^. The potential application of MSC-derived EVs in the management of various pathological conditionshas been exploredbecause these vesicles possess almost all the properties of the parent cells in terms of paracrine effects and immunomodulatory functions^24,25^.

In the present study,the potential of modulatingundifferentiated (naïve) MSCs withEVs derived from human IPCs was explored. In this manuscript, the term "extracellular vesicles" is used as recommended by the International Society for Extracellular Vesicles^26^. The term (s) used by the authors of the referenced publicationsare kept unchanged.

## Materials and methods

### Retrieval, expansion, and differentiation of human adipose tissue mesenchymal stem cells (hAT-MSCs)

The required approval for this study was obtained from the Ethical Committee of the University of Mansoura (IRB: R. 23.02. 2068). We confirm that all methods were performed in accordance with the relevant guidelines and regulations. Liposuction aspirates were obtained from 3 consenting healthy subjects during elective cosmetic surgeries. The aspirates were digested with 0.075% collagenase type I (Sigma-Aldrich, St. Louis, USA) for 30 min at 37 °C with gentle stirring. Collagenase was inactivated with an equal volume of complete low-glucose-Dulbecco’s modified Eagle’s medium(DMEM-LG, Sigma-Aldrich) supplemented with 10% fetal bovine serum (FBS),and the aspirates were centrifuged for 10 min at 300×g. The cell pellet was resuspended in complete DMEM-LG supplemented with 10% FBSand filtered through a 100 µm mesh filter to remove debris. The resuspended cells were plated at a density of 1 × 10^5^ cells/cm^2^ in 75 cm^2^ culture flasks and incubated at 37 °C in a 5% CO_2_ incubator. Three days later, the nonadherent cells were discarded. The adherent cells were cultured to 80% confluence before they were passaged with a trypsin–EDTA solution (Gibco, NY, USA). The cells were re-cultured in complete DMEM-LG, replated at a ratio of 1:2 and cultured to 80% confluence. This step was repeated for three passages. At this point, the cells were spindle-shaped and displayed a fibroblast-like appearance. Their phenotype was determined by flow cytometry and their ability to undergo trilineage differentiation into chondrocytes, adipocytes and osteocytes was tested.

At passages 3–5, cultured cells were rinsed with 1 × Dulbecco's phosphate-buffer saline (DPBS) without Mg^2+^ and Ca^2+^ (Invitrogen, Waltham, Massachusetts, USA) followed by incubation with trypsin–EDTA solution for 3 min, at 37 °C. The detached single cells were rinsed with low-glucose (1 g glucose/L) xeno-free human MSC-medium (R & D Systems, MU, USA) and centrifuged at 300×g for 5 min. The resulting cell pellet was resuspended in the same medium, seeded in laminin 521-coated flasks at 1 × 10^5^ cells/cm^2^ and cultured for 48 h (Biolamina, Stockholm, Sweden). Subsequently, the cells were cultured in serum-free DMEM-LG supplemented with 100 ng/ml activin-A (R & D Systems), 3 µM CHIR99021 (Sigma-Aldrich), 100 nM wortmannin (ENZO Life Sciences Inc., NY, USA) and 1% B-27 minus insulin (Life Technologies Corporation, NY, USA) for two days. The cells were then cultured in DMEM-LG supplemented with 100 ng/ml activin-A, 3 µM CHIR99021 and 1% B-27 minus insulin for two days and then cultured overnight in a serum-free DMEM-LG supplemented with 55 nM trichostatin-A (Sigma-Aldrich). For the next 12 days, the cells were cultured in high-glucose (4.5 g glucose/l) human MSC-medium supplemented with10 mM nicotinamide (Sigma-Aldrich), 10 nM glucagon-like peptide-1 (Sigma-Aldrich), 10 µg/l PRDX6 protein (BioVision, CA, USA) and 0.1 nM exendin-4 (Sigma-Aldrich). The media were replaced every three days. Finally, the cell suspension was seeded in ultralow adherent flasks for 3 days to enhance differentiation and to form islet-like clusters. These cell clusters were stained for insulin and C-peptide. For flow cytometry, some of these cell clusters were dissociated by trypsin. A few of these dissociated cells were cultured as a monolayer on chamber slides (Nunc, International, USA)for 24 h and then stained for GAD-65.

### EV harvesting and characterization

EVs were collected from the culture media of undifferentiated (uneducated) and differentiated (educated)hAT-MSCs. The cells were then incubated for 48 h in the same medium supplemented with EV-depleted FBS.The elimination of EVs from FBS was carried out by overnight ultracentrifugation at 100.000×g. EVs were then obtained by sequential centrifugation of the supernatant at 4°C. Initially, cell debris and large vesicles were removed by centrifugation at 300×g for 10 min, 2000×g for 20 min and 10.000×g for 30 min. Subsequently, ultracentrifugation of the supernatant was carried out at 100.000×g for 2 h. The resulting EV pellet was then washed with PBS. The EVswere diluted with PBS to obtain harvested EVs from the media of 4 X10^7^ hAT-MSCsin 1 ml. Their protein concentration was measured with a BCA protein assay kit (Millipore, Darmstadt, Germany). Finally, the specimens were aliquoted and stored at − 80 °C. EVs were characterized by TEM (Supplementary file : Data S1),dynamic light scanning for particle distribution analysis (Supplementary file : Data S2),flow cytometry for expression of specific proteins: (Supplementary file : Data S3),and Western blotting (Supplementary file : Data S4).

### Labeling of EVs and uptake by hAT-MSCs

MSC-derived EVs were stained with Exoria (Exopharm Ltd, Melbourne, Australia)as described by Tertel et al.^27^. Briefly, 1 mg of Exoria was dissolved in 1 ml of PBS. The solution was centrifuged at 17.000×g for 10 min to reduce background noise. Then 25 μl of the EV preparation was incubated for 1 h at 37 °C with 25 μl of Exoria solution. One milliliter of 0.5% serum bovine albumin was then added to bind the unbound dye. The labeledEVs were washed with PBS by ultracentrifugation at 100.000×g for 70 min. MSCs (1 × 10^5^) were cultured on Lab-Tek ll chamber slides (Nunc International, NY, USA) for 24 h at 37 °C. Ten microliter of the labeledEVs were added to the cultured cells and incubated for 24 h at 37 °C. The culture medium was then discarded, and the cells were washed 3 times with PBS. The red fluorescence signal among the hMSCs was detected by confocal microscopy to determine whether internalization of EVs into the cells had occurred.

### Cell and EV coculture

The optimal MSC/EV ratio and the duration of coculture were initially titrated to determine the optimal experimental conditions. Accordingly, a total of 1X10^5^ MSCs were treated with 80 μg of EVs/ml for 24 h at 37 °C. (Supplementary file: Data S5).The cells were then washed twice with PBS and cultured in fresh high-glucose DMEM supplemented with 10% FBS. The medium was changed every 3 days for a period of 20 days.

### Evaluation of the cell preparations

*Immunocytochemistry* The primary antibodies used were mouse monoclonal anti-insulin (Cat #8138, Cell Signaling Technology, Danvers, MA, USA), rabbit monoclonal anti-glucagon (Cat # 8233, Cell Signaling Technology), rabbit polyclonal anti-c-peptide (Cat # 4593, Cell Signaling Technology), rabbit polyclonal anti-human somatostatin (Cat # GTX39061, Gene Tex, Alton Pkwy Irvine, CA, USA) and rabbit monoclonal anti-GAD65 (Cat # 5843,Cell Signaling Technology). The secondary antibodies used were (H + L Alexa Flour 488 conjugate) anti-mouse IgG heavy and light (Cat # 4408, Cell Signaling Technology), and Alexa Flour 555 conjugate anti-rabbit IgG (H + L) (Cat # 4413, Cell Signaling Technology). The nuclei were counterstained with DAPI (Cat # 4083, Cell Signaling Technology). The cells were plated on chamber slides, and fixed with 4% paraformaldehyde for 10 min at room temperature (RT). The cells were then permeabilized with 100% chilled ethanol for 10 min, blocked with 5% normal goat serum for 60 min and incubated overnight with the primary antibodies. Subsequently, the cells were washed with PBS and incubated with the secondary antibodies for 2 h at RT.Negative controls were prepared by omitting the primary antibody and by staining undifferentiated cells. Confocal images were captured using a Leica TCS SP8 microscope (Leica Microsystems, Mannheim, Germany).

### Flowcytometry:quantification of insulin and C-peptide-positive cells

Primary monoclonal antibodies against insulin (Cat # 565688, BD, San Jose, CA, USA), and C-peptide (Cat # 565830, BD) and for secondary antibody anti mouse IgG Fab2 Alexa Flour 488 (Cat # 8878 Cell Signaling Technology) were used. The protocols used for cell preparation and labeling are detailed in Supplementary File: Data S6. Labeled cells were identified using a red laser at a wavelength of 488 nm by a FACS Aria III cell sorter (BD). A total of 20,000 events were obtained. Stained and unstained undifferentiated cells and differentiated cells processed without the primary antibody served as negative controls. The data were analyzed by Flow Jo software (Becton, Dickinson).

*Nanogoldimmunostaining* The cells were fixed in 2% formalin and 0.5% glutaraldehyde (both EM grade from Ted Pella) in phosphate-buffered saline (PBS) overnight at 4°C. After washing 3 × 15 min with phosphate-buffered saline (PBS) containing 0.05% Tween 20 (Bio-Rad Laboratories, Hercules, CA, USA), they were blocked for 6 h in casein blocking buffer (BioRad) with 0.05% Tween 20 at room temperature. Subsequently samples were incubated with anti-C-peptide antibody (Cat# 05-1109, MilliporeSigma, Burlington, MA, USA) at 1:50 dilution in blocking buffer, overnight at 4 °C. The next day, samples were washed for several hours with PBS-0.05% Tween 20 and incubated overnight at 4 °C with anti-mouse antibody conjugated with 1.4 nm gold Cat #2001; Nanoprobes, Yaphank, NY, USA) diluted at 1:50 with blocking buffer. The next day, samples were washed 3 × 15 min with PBS-0.05% Tween 20, post-fixed for 20 min in 1% glutaraldehyde in PBS-0.05% Tween 20, washed 3 × 15 min in water, silver enhanced with the silver enhancement kit (Cat #2013, Nanoprobes) and processed for electron microscopy without the osmium tetroxide treatment, as described previously^28,29^. Thin (70 nm) sections were examined and photographed in JEOL 1200 transmission electron microscope. Undifferentiated MSCs as well as human islet were similarly labeled to serve as negative and positive controls respectively.

### Gene expression by real-time PCR

Total RNA was extracted from undifferentiated cells, cells at the end of in vitro differentiation and cells following coculture with EVs using a Direct-Zol™ RNA Miniprep kit (Zymo Research, California, USA). The RNA concentration was measured with a spectrophotometer (Nanodrop 2000, Thermo Fisher Scientific, Massachusetts, USA). Thereafter, three micrograms of total RNA were converted into cDNA using an RT2 First Strand Kit (Qiagen Sciences, Germantown, MD, USA). Primers were designed using the website of the National Centre for Biotechnology Information.In this study, the expression of relevant pancreatic endocrine genes was evaluated. The expression of the following genes was determined: the pancreatic endocrine hormones insulin (INS), glucagon (GCG), and somatostatin (SST); the relevant transcription factors pancreatic and duodenal homeobox 1 (PDX1), neurogenin3 (NGN3), regulatory factor X6 (RFX6), neurogenic differentiation factor 1 (NEUROD1), V-Maf musculoaponeurotic fibrosarcoma oncogene A and B (MAFA & MAFB) and paired box 4 (PAX4); the pancreatic enzymes: glucokinase (GCK); the glucose transporter solute carrier family member 2 (GLUT-2); the endocrine precursor marker nestin (NES); and the nuclear hormone receptor superfamily member estrogen-related receptor gamma (ESRRɣ). Glyceraldehyde-3-phosphate dehydrogenase (GAPDH) was included as an internal control for normalization. Amplification was performed for each sample in a 20 µl reaction consisting of 10 µl of 2X Maxima SYBR Green Master Mix (Thermo Fisher Scientific), 2 µl of primers (5 pmol), 1 µl of cDNA template (100 nmol), and 7 µl of nuclease-free water. The reactions were carried out in a 96-well plate inserted into a real-time thermal cycler (CFX96 Real-Time System, Bio—Rad, Hercules, CA, USA). The cycling parameters for PCR amplification were as follows: initial denaturation at 95 °C for 3 min, followed by 40 cycles of denaturation at 95 °C for 15 s, annealing at 60 °C for 30 s and extension at 72 °C for 30 s. The procedure was performed in triplicate for each sample. In this study, the data are expressed relative to those obtained for undifferentiated MSCs.

### Determination of in vitro insulin and C-peptide release in response to increasing glucose concentrations

Six samples, 1 million cells each, were obtained from each of the 3 cell types: MSCs differentiated by the conventional protocol (IPCs),naïveMSCs cocultured with educated EVs, and naïve MSCs cocultured with uneducated EVs. The cells were initially incubated for 3 h in glucose-free Krebs–Ringer bicarbonate buffer (KRB). This step was followed by incubation for 1 h in 3.0 ml of KRB containing 5.5, 12 or 25 mM glucose. The supernatant was collected at the end of each incubation period. The collected samples were frozen at − 70 °C until assayed using an ELIZA Kit for human insulin (DRG Diagnostic, Germany) and for human C-peptide (Abcam, Cambridge, UK).

### Statistical analysis

Data analysis was carried out using IBM SPSS statistics 16.0 software (IBM Corp., Armonk, NY, USA). According to their category, the data are expressed as median values or as the mean ± standard error. Since the data were nonparametric and unmatched, significant differences between 2 groups were analyzed by the Mann–Whitney test. For more than 2 groups, Kruskal–Wallis 1-way analysis of variance was used. A *p* value of < 0.05 was considered significant.

### Ethics approval and consent to participate

The required approval for this study (Modulation of naïve mesenchymal stromal / stem cells by extracellular vesicles derived from insulin-producing cells: an in vitro study) was obtained from the Institutional Research Board of the University of Mansoura (IRB: R. 32.02.2068 / date 26-2-2023). The human adipose donors gave written informed consent for the use of their samples in this study (without a financial compensation).

## Results

### Characteristics of the MSCs

At the end of expansion, the MSCs adhered to the plastic of the culture plates, exhibited a spindle-shaped morphology, and were positive for their specific cell markers and negative for the hematopoietic stem cell markers (rawdata, Supplementary file: S7). In addition, their abilityfor trilineage differentiationinto adipocytes, chondrocytes and osteocytes was verified (Supplementary file: S8).

### Characteristics of the isolated EVs (Fig. 1)

**Figure 1:**
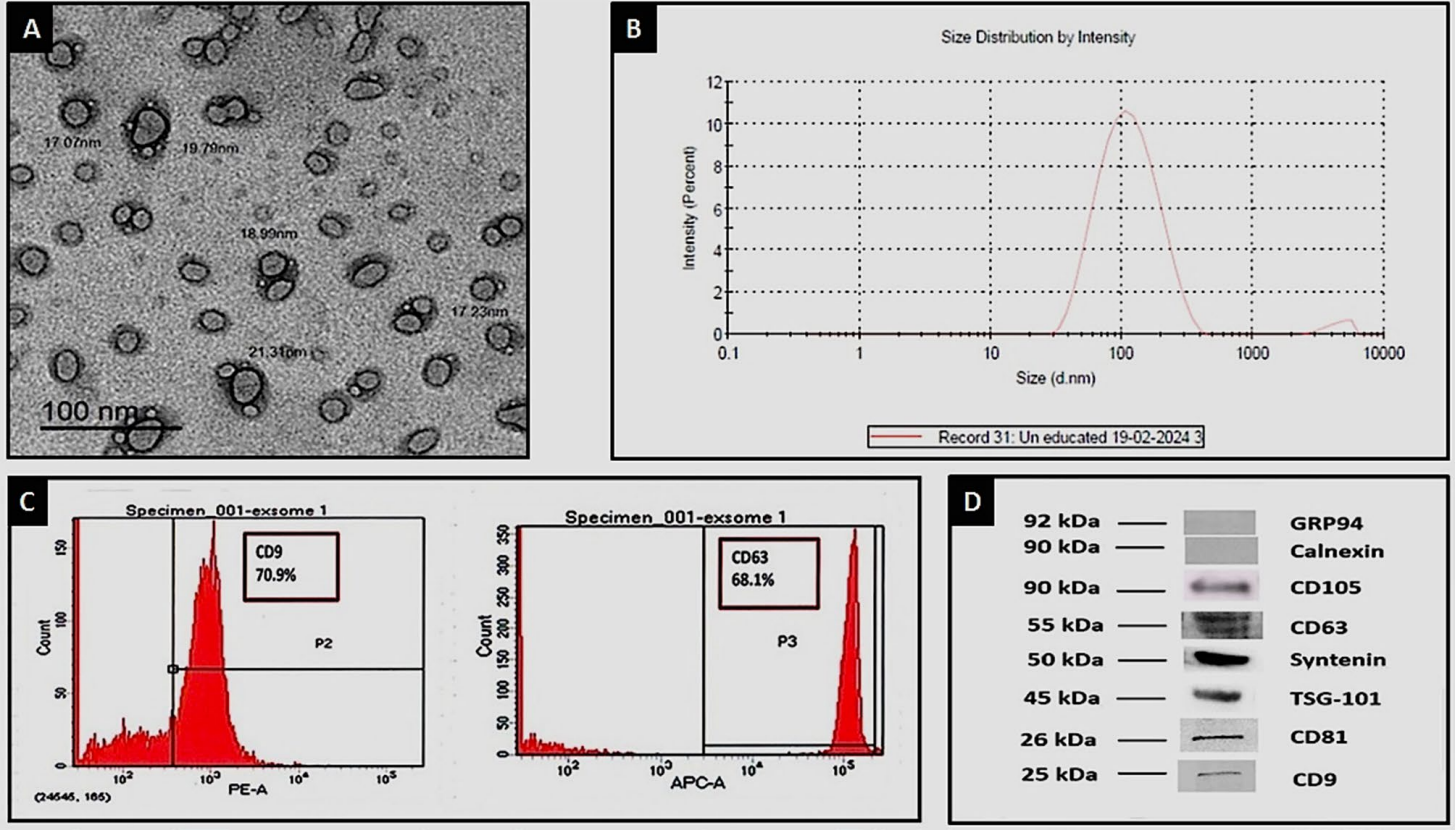
Characterization of the isolated EVs: (**A**) TEMshows multiple spherical vesicular EVs. (**B**) Particle size distribution by intensityanalysis showed that the largest aggregate of EVs (97%) had an average diameter of 123.8 nm. (**C**) Flowcytometry confirmed that the isolated EVs expressed CD9 and CD63. (**D**) Western blotting confirmed that the isolated EVsexpressed CD81, CD9, CD63, syntenin and TSG-101 and did not express GRP94 or calnexin. The expression of CD105 confirmed the MSC-origin of these EVs. Uncropped gel blots are included in supplementsry file: S12.

The spherical cup-shaped morphology of MSC-derived *EVs* was confirmed by TEM (Fig. 1A). Particle distribution analysis by intensity showed that the largest EV-aggregate (97.0%) had an average diameter of 123.8nm (Fig. 1B). A second and much smaller cluster of apoptotic bodies with an average diameter of > 1000 nm was also noted. The expression of the specific associated proteins CD9, and CD63 was verified by flowcytometry (Fig. 1C), and that of CD9, CD63, CD81, TSG-101 and syntenin was verified by Western blotting (Fig. 1D).

### Internalization of the isolated EVs into MSCs

**Figure 2:**
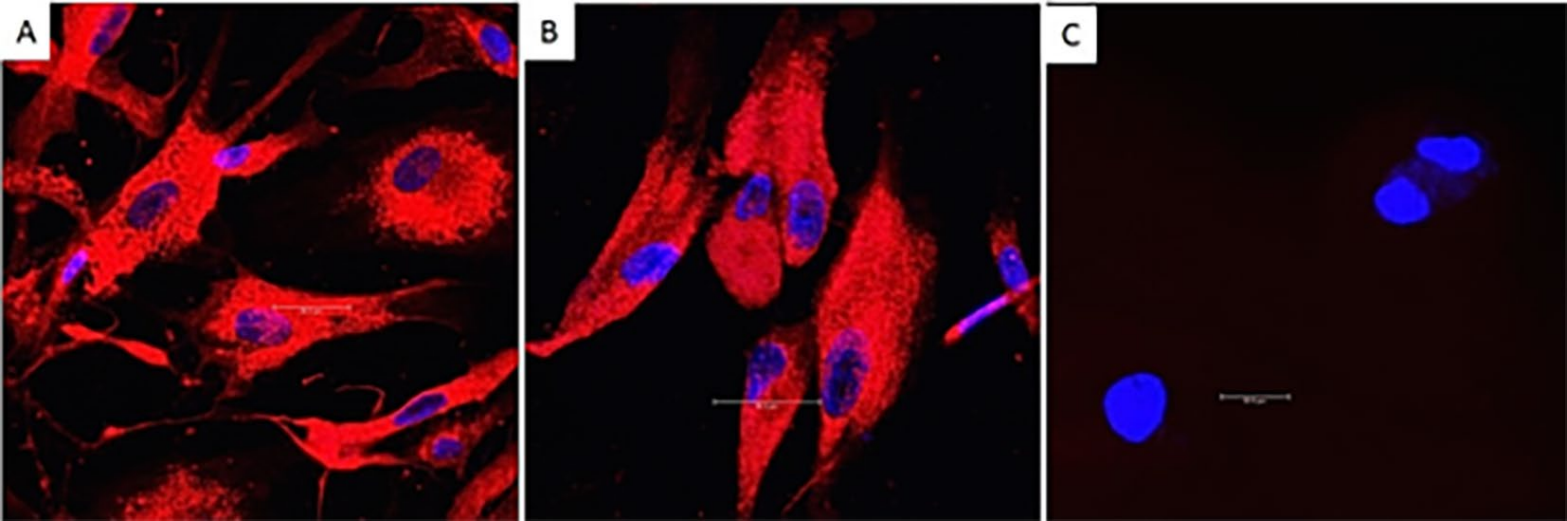
EVs (educated and uneducated) were incubated with red Exoria dye. The red-labeled EVs were then incubated with MSCs. Internationalization of EVs wasconfirmed when the cells acquired a red stain. (**A**) MSCswere incubated with Exoria-labeled educated EVs. Red staining indicates that educated exosomes were internalized into MSCs. (**B**) MSCswere incubated with Exoria-labeled uneducated EVs. Red staining indicated that uneducated exosomes were internalized intoMSCs. (**C**) The internalization of unlabeled exosomes served as a negative control. The cells lack the red color, and their nuclei are stained blue with DAPI.

Coculture of Exoria-labeledEVswith MSCs confirmed the internalization of the EVs (Fig. 2).

### Immunocytochemistry for insulin and C-peptide (Fig. 3)

**Figure 3:**
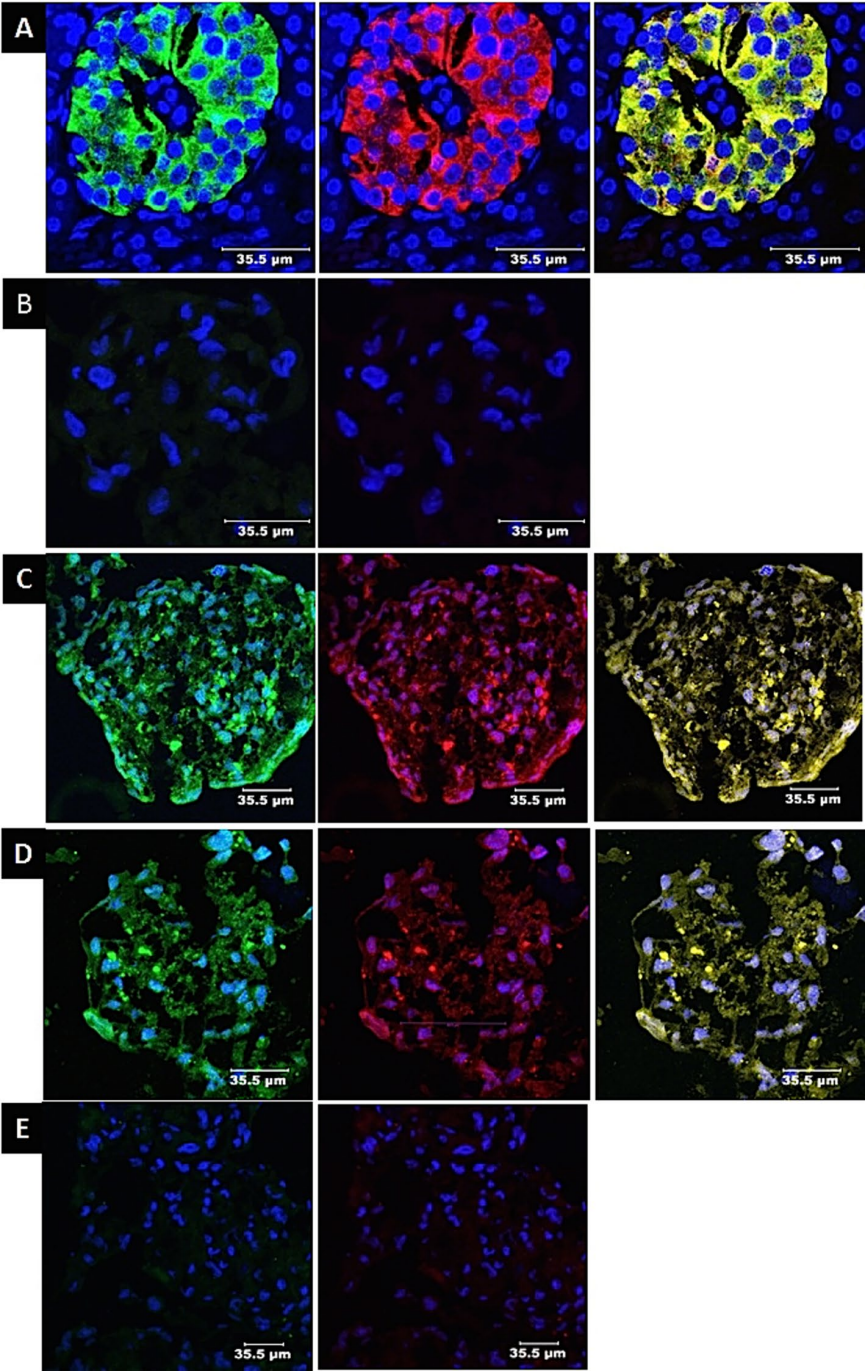
Immunocytochemical staining for pancreatic hormones: (**A**) Positive control. Immunostaining of a human pancreatic islet. Positive staining for insulin (green), and C-peptide (red). A merged image of insulin and C-peptide (yellow) indicates co-expression of the two molecules within the same cells. (**B**) Negative control. Immunostaining of naïve MSCs. The cells were negative for insulin and C-peptide. Only blue nuclei stained with DAPI are visible. (**C**) Differentiated IPCs. The cells were positive for insulin (green) andC-peptide (red). A merged image of insulin and C-peptide (yellow),indicates that insulin and C-peptide are expressed within the same cells. (**D**) Naïve MSCs were cocultured with educated EVs. Cells are positivefor insulin (green) andC-peptide (red). A merged image of insulin and C-peptide cells (yellow), indicates the coexpression of these two molecules within the same cells. (**E**) Naïve MSCs were cocultured with uneducated EVs. The cells were negative for insulin and C-peptide. Only blue DAPI stained nuclei were visible.

Insulin and C-peptide were co-expressed in human islets (Fig. 3A), MSC-differentiated IPCs (Fig. 3C) and naïve MSCs cocultured with educated EVs (Fig. 3D). In contrast, naive MSCs (Fig. 3B) and naïve MSCs cocultured with uneducated EVs (Fig. 3E) did not express insulin or C-peptide.

### Immunocytochemistry for GAD65 (Fig. 4)

**Figure 4:**
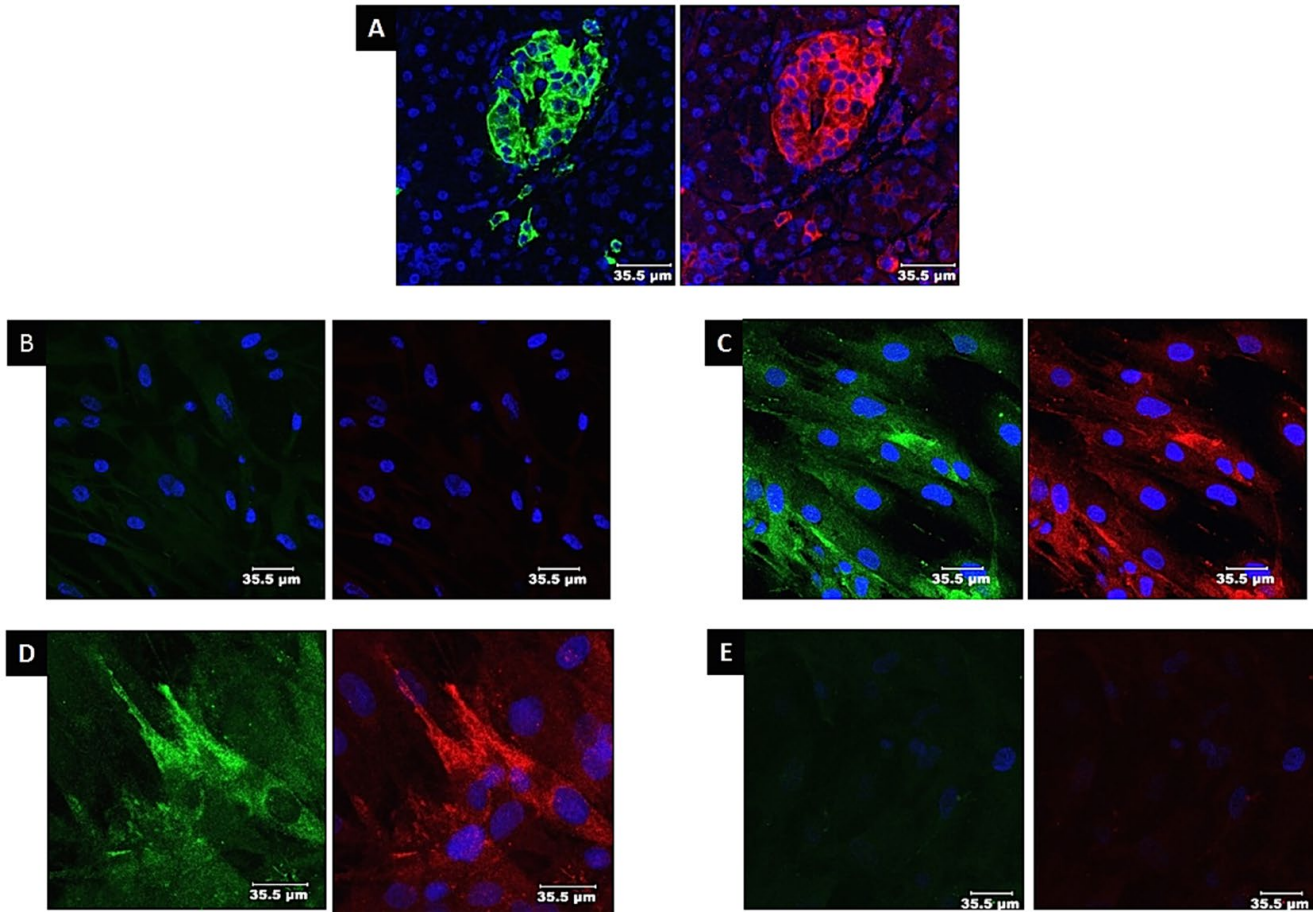
Immunostaining for GAD65: (**A**) Positive control: human islets. Cells are positive for insulin (green) and GAD65 (red). (**B**) Naïve MSCs: The cells did not express insulin or GAD65. Only blue DAPI-stained nuclei were visible. (**C**) Differentiated MSCs (IPCs): The differentiated IPCs are positive for insulin (green) and GAD65 (red). (**D**) Naïve MSCs were cocultured with educated EVs: Cells expressed insulin (green) and GAD65 (red). (**E**) Naïve MSCs cocultured with uneducated EVs: The cells were negative for insulin and GAD65. Only blue-DAPI stained nuclei are visible.

GAD65 was expressed in human islets (Fig. 4A), MSC-differentiated IPCs (Fig. 4C) and naïve MSCs cocultured with educated EVs (Fig. 4D). naïve MSCs (Fig. 4B) and MSCs cocultured with uneducated EVs (Fig. 4E) did not express GAD65.

### Quantification of the hormone-positive cells by flow cytometry (Fig. 5)

**Figure 5:**
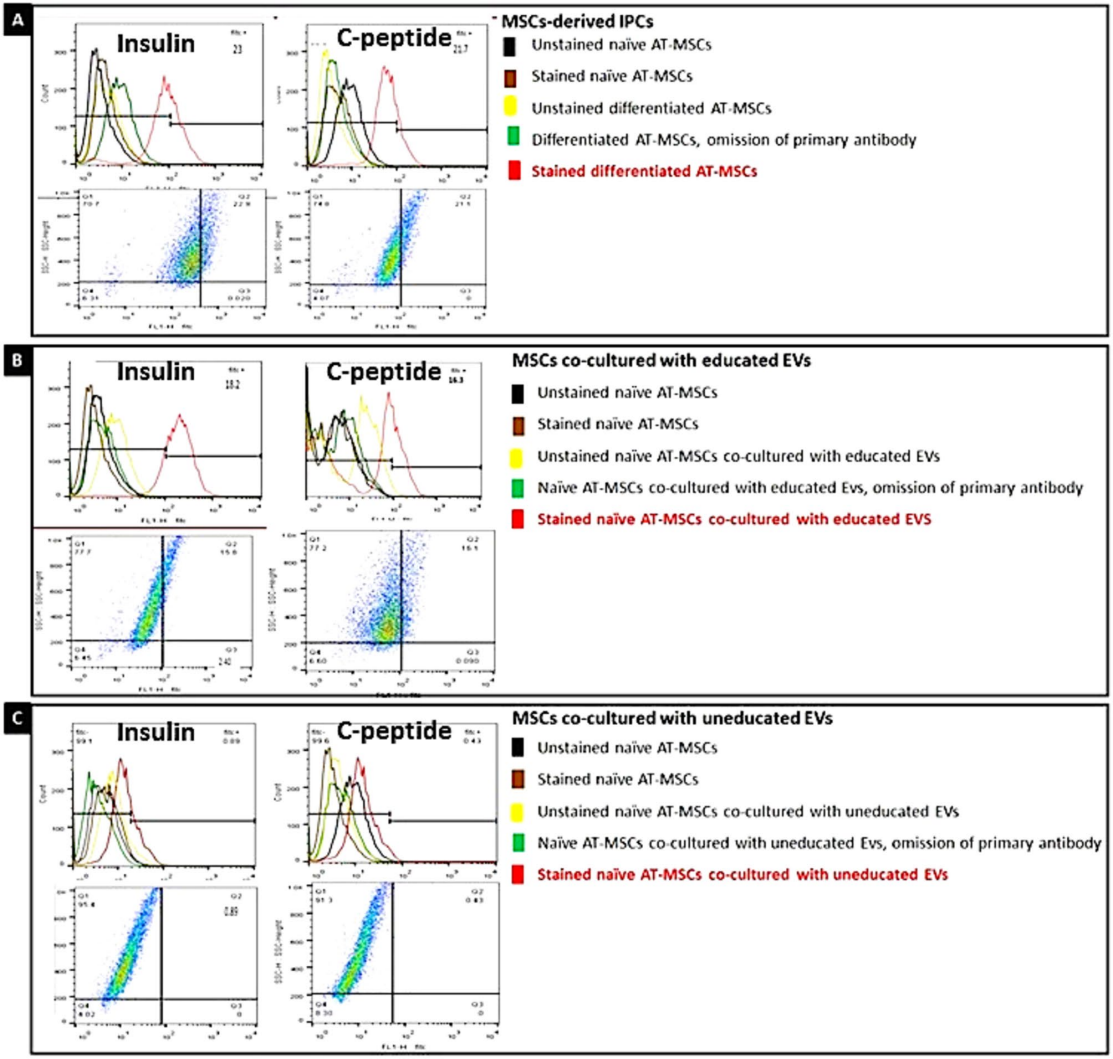
Quantification of hormone-positive cells by flow cytometryin an example study: (**A**) MSCs differentiated into IPCs. The percentage of insulin-positive cells was 23% and that for C-peptide-positive cells was 21.7%. (**B**) Naïve MSCs were cocultured with educated EVs. The percentage of insulin-positive cells was 18.2% and that for C-peptide positive cells was 16.3%. (**C**) Naïve MSCs were cocultured with uneducated EVs. The percentage of insulin-positive cells was 0.89% and that for C-peptide positive cells was 0.43%.

The median percentages of insulin-and C-peptide-positive cells among the differentiated IPCs (donor cells) were 21.2% and 18.2% respectively. The median percentages of insulin-and C-peptide-positive cells among the MSCs cocultured with educated EVs (recipient cells) were 16.15% and 14.25% respectively. In contrast the median percentages of insulin-and C-peptide-positive cells among MSCs cocultured with uneducated EVs were 2.05% and 0.77% respectively. (raw data, Supplementary file: S9). These data represent the median of 6 experiments. Figure 5 and its legend represent an example case.

### C-peptide nanogold, silver-enhanced, immunostaining (Fig. 6)

**Figure 6:**
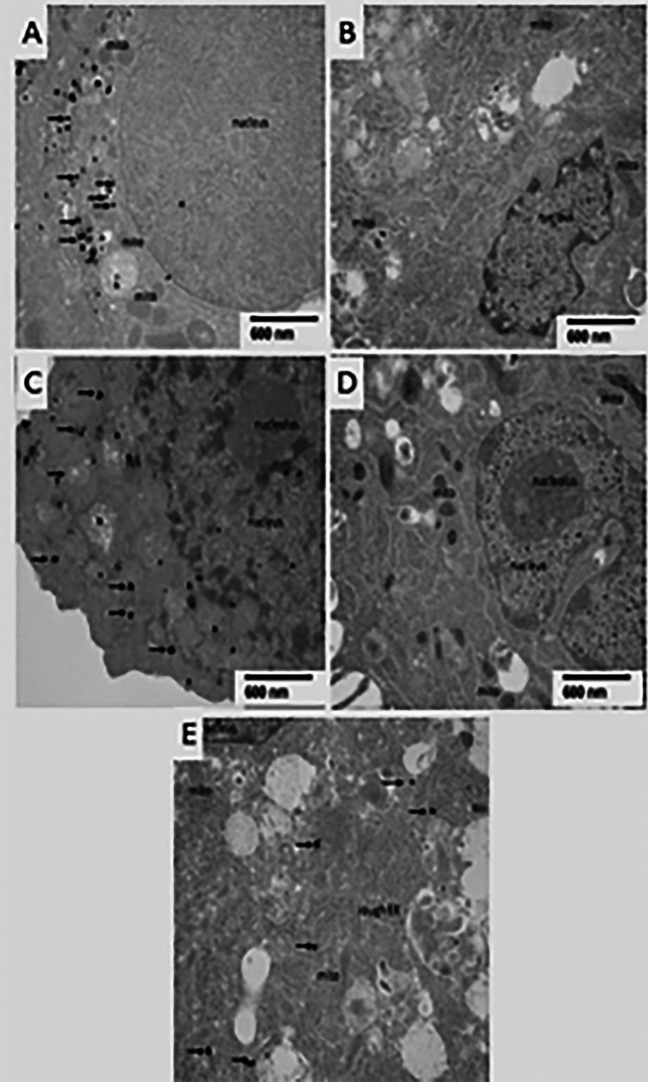
TEM. Silver enhanced C-peptide nanogold immunostaining: (**A**) Pancreatic islet cell contains labeled c-peptide (black spots,arrows) in the cytoplasm. (**B**) Naïve MSC negative for C-peptide. (**C**) Differentiated MSC contains labeled C-peptide (blackspots,arrows) in the cytoplasm. (**D**) Naïve MSC cocultured with uneducatedEVs is negative for C-peptide. (**E**) Naïve MSC cocultured with educated EVsshows labeled C-peptidein the cytoplasm (black spots,arrows). Mitochondria (mito), rough endoplasmic reticulum (RER). To visualize the silver enhanced nanogold particles the TEM samples were not contrasted with osmium, thus the ultrastructural features of cells are not well visible.

Pancreatic islet cells contained labeled C-peptide (arrows) in the cytoplasm. Undifferentiated MSCs were negative for C-peptide while differentiated IPCs containedlabeled C-peptide in the cytoplasm. Undifferentiated MSCs cocultured with uneducated EVs were negative for C-peptide. In contrast undifferentiated MSCs cocultured with educated EVs showed labeled C-peptide (arrows) in the cytoplasm. To visualize the silver enhanced nanogold particles, TEM samples were not contrasted with osmium, thus the ultrastructural features of cells were not well visible.

### Gene expression by RT-PCR (Fig. 7)

**Figure 7:**
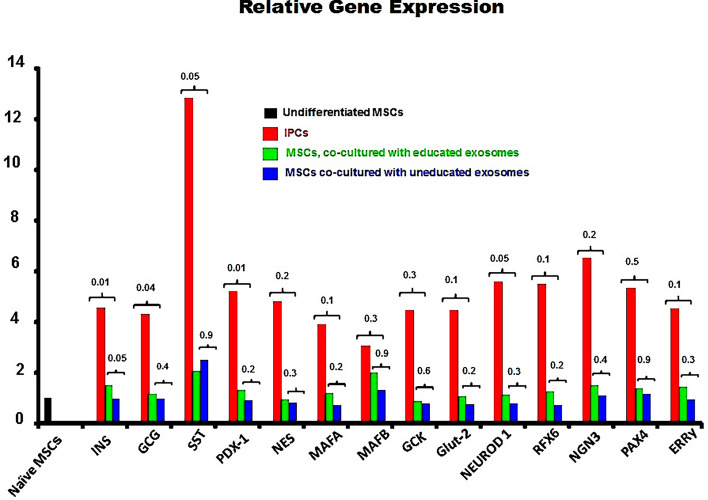
Relative gene expression determined by RT-PCR. (median values of 8 studies): All pancreatic endocrine genes were expressed by differentiated IPCs and the naïve MSCs cocultured with educated EVs. The expression of INS, GCG, SST, PDx-1 and NEUROD-1 were significantly greater in IPCs. Gene expression in naïve MSCs cocultured with uneducated EVs was marginal.

All the relevant pancreatic endocrine genes were expressed by thedifferentiated IPCs. The expression levels were greater than those of MSCs cocultured with educated or uneducated EVs. Differences in the expression of *INS, GCG, SST, PDX-1 and NEUROD-1*were significant. Gene expression values among MSCs cocultured with educated or uneducated EVs were comparable. A significant difference was only noted for the expression of insulin(raw data, Supplementary file: S10).

### Insulin and C-peptide release in response to increasing glucose concentrations (Fig. 8)

**Figure 8:**
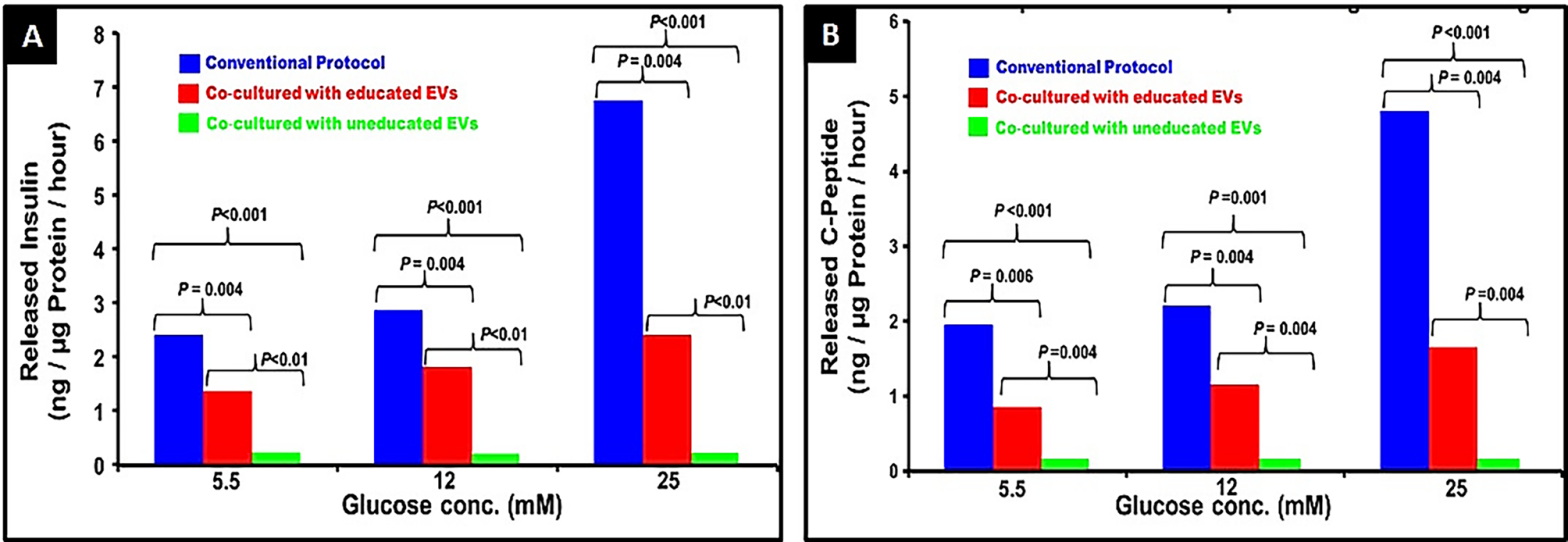
Insulin and C-peptide release in response to increasing glucose concentrations: There was a stepwise increase in the amount of insulin and C-peptide released from differentiated IPCs and naïve MSCs cocultured with educated EVs. The values of the released insulin and C-peptide were significantly greaterfor the IPCs. The responses of MSCs cocultured with uneducated EVs were negligible.

There was a stepwise increase in the amount of insulin and C-peptide released from differentiated IPCs and naïve MSCs cocultured with educated EVs. There was a statistically significant increase in the secretion of human insulin and C-peptide in response to the glucose challenge at each concentration (raw data, supplementary file 1: S11). The release of insulin and/or C-peptide by MSCs cocultured with uneducated EVs in response to glucose challenge was negligible. (raw data, Supplementary file: S11).

## Discussion

Although MSC-based therapies are considered safe^30^, their systemic administration carries some inherent risks. Cells are trapped in the lungs and can lead to the occlusion of the microvasculature. The ability of MSCs to differentiate into chondrocytes and osteocytes has raised concernsabout undesirable ectopic calcification or ossification in tissues^31^. Transplantation of living dividing cells carries a risk of teratogenesis. Cell necrosis and/or the induction of allogenic immune responses are additional challenges. In contrast, because of their nanosize, EVs can be safely infused without being trapped in the lungs or occlusion of the microvasculature.EVs do not replicate, and the potential for teratogenesis is eliminated^32^. The production of EVs is scalable, and these vesicles can be stored and used as off-the-shelf therapeutic tool and can then be delivered on a timely basis^33,34^. The donor cell can be colonially selected and immortalized to ensure reproducible production^35^. Since MSC-derived EVs exert the same functions as their parent cells, their use is safer and more economical than cell-based therapeutics. As a result, the use of EVs for the treatment of several disease entities has been increasingly reported^24,25,36–38^.

The main objective of our in vitro study is to determinewhether IPC-derived EVs can modulate naïve MSCs into IPCs. To this end, hAT-MSCs were isolated, expanded and characterized by their morphology, phenotype and ability to undergo trilineage differentiation. The cells were then differentiated into IPCs by our conventional protocol^39^. Uneducated EVs were retrieved from the conditioned medium of naïve MSCs, and educated EVs from that of IPCs. Harvesting was carried out by differential centrifugation without preliminary steps; an approach that has long been considered the most efficient and popular method to isolate EVs from cell cultures^40^.To date, there are no standardized protocols for eliminating EVs from FBS. Overnight ultracentrifugation at 100,000×g was used and adopted in our protocol^41^.EVs were then fully characterized according to the recommendation of the International Society for Extracellular Vesicles^26^. A spherical cup-shaped morphology was observed by TEM. The particle-size analysis was performed by dynamic light scattering with an emphasis on measurement by intensity. The different types of distributions can often produce substantially different results. The intensity data are closest to what is actually measured since they indicate how much light is scattered by particles in different size bins^42^. In our study, EVs with an average diameter of 124 nm were obtained. The presence of specific proteinsin the retrieved EVs was confirmed by flow cytometry and Western blotting. The presence of CD105 reflected their MSC-origin. EV internalization was confirmed by coculture of Exoria-labeled EVs with MSCs. According to Tertel et al. Exoria was the only dye that specifically labeledEVs in MSC-EV preparations^27^. Uneducated and educated EVs were cocultured with naïve MSCs. An immunofluorescence study demonstrated that insulin- and C-peptide-positive cells were only obtained from IPCs and naïve MSCs cocultured with educated EVs. Unexpectedly, IPCs and naïve MSCs cocultured with educated EVs stained positive for GAD65, while cells cocultured with uneducated EVs did not. This finding suggests that the IPCs and/or the modulated cells can be the subject of destruction by autologous antibodies responsible for T1DM. Can their immunomodulatory function overcome this problem? A question that needs further investigations in a T1DM animal model. Hormone-positive cells werequantified by flow cytometry. We relied on C-peptide values since they are more accurate than insulin-based measurements. Insulin present in the culture media can be absorbed and sequestered in the cells resulting in false higher readings^43^. The median percentages of C-peptide- positive cells were 18.2% and 14.2% among IPCs and naïve MSCs cocultured with educated EVs respectively. These levels are sufficient to provide functional benefits since cell transplantation is followed by further differentiation in vivo^44^. Again, immunogold labeling revealed C-peptide-positivegranules within IPCs and naïve MSCs cocultured with educated EVs. A stepwise increase was observed in the amount of insulin and C-peptide released in response to glucose challenge amongIPCs and naïve MSCs cocultured with educated EVs. This finding indicates that cells from both categories are glucose-sensitive and insulin-responsive. The responses of cells cocultured with uneducated EVswere negligible. Overall, evidence for the intrinsic synthesis of insulin and C-peptide by naïve MSCs cocultured with educated EVs was provided and their modulation into IPCs was established.

The results of and the acquired benefits from administration of uneducated MSC-derived EVs in diabetic animal models were the subject of previous reports. In all these experimental studies areduction in fasting glucose levels was reported, but euglycemia was not achieved. This therapeutic benefit was attributed to a variety of factors. EV immunomodulatory functions^45,46^, promotion of autophagy^47^, and increased expression of genes associated with regeneration^48^, have been suggested. Sun et al. proposed that treatment with exosomes restored the phosphorylation of insulin receptor 1, promoted the expression of glucose transporter 4 and increased glycogen storage in muscles^49^. The transfer of an insulinotropic factor enclosed within the EV-cargo to recipient cells was proposed by Kulaj et al.^50^. Alternatively, the use of educated exosomes derived from the conditioned medium of β cells has also been reported. Sun et al. harvested EVs from an insulinoma cell line (MIN6). Diabetes was chemically induced in male C57BL/6J mice. EVs were transplanted into the pancreas. The authors observed that the treated animals had a longer median survival time than the controls. Improved glucose tolerance and increased insulin content with preservation of islet architecture were detected. The expression of CD31, a marker of endothelial cells, was also enhanced^51^. Guo et al., cocultured exosomes derived from the MIN6 cell line with human induced pluripotent stem cells (iPSCs). Compared with the controls, the treated iPSCs showed greater expression of the relevant pancreatic endocrine genes and were positively stained for insulin and glucagon by immunofluorescence. The expression of these pancreatic markers was significantly reduced in the iPSCs cocultured with EVs derived from MIN6 cells pretreated with silenced Agonaute2 (siAgo2). The EV-induced iPSCs were transplanted under the renal capsule of STZ-diabetic mice. Glucose tolerance was improved and a 50% decrease in blood glucose levels was noted. Dynamic changes in the expression of 4 miRNAs:miR-706, miR-709, miR 466c-5p and miR-423-5p were detected in the treated iPSCs. Based on this finding, the authors maintained that βcell-derived EVs induce the differentiation of iPSCs into IPCs via miRNA-dependent mechanisms^52^. Bai and associates used a 4-stage procedure for chemical differentiation of human iPSCs. EVs derived from human β cells were added in stage IV^53^.The resulting induced β cells (i-β cells) secreted insulin in response to a glucose challenge. Transplantation of these i-β cells under the renal capsule of STZ-induced diabetic mice resulted in a significant reduction in their blood glucose levels. Functional analysis of these EVs revealed that 5 miRNAs were involved in the development of i-β cells from iPSCs. The authors demonstrated that miR-212/132 are the most relevant since the insulin-positive cell population was significantly decreased after inhibitors of these molecules were added. EV-miR-212/213 serve to stabilize NGN3 expression which promotes the differentiation of endocrine cells from iPSCs. Mandal and colleagues used a 3-stagechemical-based protocol for the differentiation of mouse embryonic fibroblasts into β-like cells^54^. Exosomes derived from the MIN6 cell line were added to the cell culture in stage I. The differentiated cells overexpressed the endocrine genes Pdx1 and Ngn3 and released C-peptide after challenge with a high glucose concentration. Furthermore, the authors identified the miRNA profile of MIN6-derived EVs. These researchers observed that miR-486, miR-127, miR-196, miR-494 and miR-709 were highly enriched in these EVs. Individual transfection to identify the miRNAs that induced the highest levels of Pdx1 in the recipient cells was carried out. The authors reported that miR-127 and miR-709 induced the highest Pdx1 expression.Notably, the utilized methods and the involved mechanisms were not uniform. In some reports, EVs were retrieved from an insulinoma cell line that is biologically different from human β cells^55,56^. The recipient cells were also different; pancreatic cells, human iPSCs and mouse fibroblasts were used. After transplantation, the resulting cells improved glucose tolerance and reduced blood glucose levels, but euglycemia was not achieved. However, the essential aim is to assess the outcome following systemic administration of EVs and not after transplantation of EV-cocultured cells. In our experiments, EVs were obtained from the conditioned medium of hAT-MSC-derived IPCs. Furthermore, these EVs were cocultured with MSCs. The utilization of EVs of MSC-origin and their coculture with MSCs can provide a distinct advantage. This method would exploit the benefits of the immunomodulation and pro-angiogenesis functions of these cells.

Due to their complex composition, EVs can deliver different bioactive molecules to recipient cells that can modify their function and phenotype^21^. Various mechanisms have been proposed to explain their mode of action. Early pioneering studies by Ratajczak and Valedi showed that the functional mRNA transferred to recipient cells can be translated to protein^18,19^. This hypothesis was supported by several investigators^8,57–59^. EVs can also transfer miRNAs to target cells^60,61^. Collino et al., reported that microvesicles released from human MSCs contain miRNAs that are more abundant than in the cell of origin suggesting specific and selective compartmentalization^62^. EV-mediated transfer of miRNAs regulate protein translation and modulate the expression of gene products in recipient cells^63^. The transfer of genetic information was questioned by Toh and associates^64^. They argued that EV-RNAs are too short to carry protein coding information. The miRNA-induced biological effects of MSC-EVs can be exerted only by either pre-miRNA or RNA-induced silencing complex (RISC)-loaded mature miRNA. These authors challenged the role of miRNA because the RISC components are not generally present in EVs. Furthermore, pre-miRNAs are not found in sufficient quantities to elicit a relevant biological response. A protein-based mode of action was proposed. More than a thousand proteins have been identified in the EV-contents. These proteins are involved in many key biological activities that are essential for cellular communication and the modulation of cell functions^65^. It was suggested that proteins in typical MSC-derived EVs are present at sufficient functional levels to elicit biological responses. In our study,despite the evidence for the intrinsic synthesis of C-peptide and insulin, gene expression was modest. We hypothesized that this functional result is due to mechanisms other than the upregulation of relevant genes.

Although experimental studies reporting the use of EVs for the treatment of DM in rodents are plentiful, clinical trials are scarce and thus far non-informative. Treatment of DM with EVs can involve 2 approaches. Uneducated EVs can be used for cases with early-onset T1DM to exploit their immunomodulatory functions. For patients without a meal-stimulated C-peptide response, the use of educated EVs can also be explored. In any event, before embarking on a clinical trial, several challenges have to be addressed. The best source for MSC-derived EVs has yet to be identified. The method for their isolation must be optimized and standardized. Increases in the yield of EVs from the conditioned medium of MSCs and their disease-specific potential are needed. The optimal dose for systemic administration, the frequency of repeat treatments, and the duration of a possible therapeutic benefit have to be determined. The organ distribution of the administrated EVs and the type of cells in which they are localized have also to be identified.The side effects of EV treatments particularly in the long term should also be noted. To address all these issues. Rigorous in vivo studies in a diabetic animal model have to be carried out.

If these experiments provide an answer for all these issues and systemic administration of educated EVs can achieve euglycemia in a diabetic animal model, the efficiency of this modality has to be compared with pluripotent stem cell-derived islets or neonatal pig islet transplantation. In addition, the results should be comparaple to or better than those of the newly approved anti-diabetic medications or the ever-improving closed-loop insulin pumps.

## Conclusions

EVs derived from insulin producing cells can modulate naïve MSCs to become insulin secreting. As yet, the mechanisms involved have not been precisely identified. The real test for the efficiency of these EVs is the ability to treat diabetes in a rodent model following their systemic administration. The required dose and frequency of their administration have to be determined. Any side effects have also to be noted. To this end, experiments in our research laboratory are underway.

### Supplementary Information

Supplementary Information.

## Data Availability

All data generated or analysed during this study are included in this published article and its supplementary information files.

## References

[1] Global status report of noncommunicable disease. Geneva: World Health Organization.(2014) https://www.who.int/publications/i/item/9789241564854.

[2] Roglic, G. WHO Global report on diabetes: A summary. *Int. J. Noncommun.* **1**, 3–8. 10.4103/2468-8827.184853 (2016). 10.4103/2468-8827.184853

[3] Shapiro, A. M., Pokrywczynska, M. & Ricordi, C. Clinical pancreatic islet transplantation. *Nat. Rev. Endocrinol.* **13**, 268–277. 10.1038/nrendo.2016.178 (2017). 27834384 10.1038/nrendo.2016.178

[4] Koro, C. E., Bowlin, S. J., Bourgeois, N. & Fedder, D. O. Glycemic control from 1988 to 2000 among U.S. adults diagnosed with type 2 diabetes: A preliminary report. *Diabetes Care* **27**, 17–20. 10.2337/diacare.27.1.17 (2004). 14693960 10.2337/diacare.27.1.17

[5] Toma, C., Wagner, W. R., Bowry, S., Schwartz, A. & Villanueva, F. Fate of culture-expanded mesenchymal stem cells in the microvasculature: In vivo observations of cell kinetics. *Circ. Res.* **104**, 398–402. 10.1161/CIRCRESAHA.108.187724 (2009). 19096027 10.1161/CIRCRESAHA.108.187724 PMC3700384

[6] Gnecchi, M. *et al.* Paracrine action accounts for marked protection of ischemic heart by Akt-modified mesenchymal stem cells. *Nat. Med.* **11**, 367–368. 10.1038/nm0405-367 (2005). 15812508 10.1038/nm0405-367

[7] Goolaerts, A. *et al.* Conditioned media from mesenchymal stromal cells restore sodium transport and preserve epithelial permeability in an in vitro model of acute alveolar injury. *Am. J. Physiol. Lung. Cell Mol. Physiol.* **306**, 975–985. 10.1152/ajplung.00242.2013 (2014). 10.1152/ajplung.00242.2013 PMC4042188 24682451

[8] Bruno, S. *et al.* Mesenchymal stem cell-derived microvesicles protect against acute tubular injury. *J. Am. Soc. Nephrol. JASN* **20**, 1053–1067. 10.1681/ASN.2008070798 (2009). 19389847 10.1681/ASN.2008070798 PMC2676194

[9] Lai, R. C. *et al.* Exosome secreted by MSC reduces myocardial ischemia/reperfusion injury. *Stem. Cell. Res.* **4**, 214–222. 10.1016/j.scr.2009.12.003 (2010). 20138817 10.1016/j.scr.2009.12.003

[10] Raposo, G. & Stoorvogel, W. Extracellular vesicles: exosomes, microvesicles, and friends. *J. Cell Biol.* **200**, 373–383. 10.1083/jcb.201211138 (2013). 23420871 10.1083/jcb.201211138 PMC3575529

[11] Lai, R. C., Yeo, R. W. & Lim, S. K. Mesenchymal stem cell exosomes. *Semin. Cell Dev. Biol.* **40**, 82–88. 10.1016/j.semcdb.2015.03.001 (2015). 25765629 10.1016/j.semcdb.2015.03.001

[12] Lötvall, J. *et al.* Minimal experimental requirements for definition of extracellular vesicles and their functions: A position statement from the International Society for Extracellular Vesicles. *J. Extracell. Vesicles* **3**, 26913. 10.3402/jev.v3.26913 (2014). 25536934 10.3402/jev.v3.26913 PMC4275645

[13] Börger, V. *et al.* Mesenchymal stem/stromal cell-derived extracellular vesicles and their potential as novel immunomodulatory therapeutic agents. *Int. J. Mol. Sci.* **18**, 1450. 10.3390/ijms18071450 (2017). 28684664 10.3390/ijms18071450 PMC5535941

[14] Vlassov, A. V., Magdaleno, S., Setterquist, R. & Conrad, R. Exosomes: current knowledge of their composition, biological functions, and diagnostic and therapeutic potentials. *Biochim. Biophys. Acta.* **1820**, 940–948. 10.1016/j.bbagen.2012.03.017 (2012). 22503788 10.1016/j.bbagen.2012.03.017

[15] Redzic, J. S., Balaj, L., van der Vos, K. E. & Breakefield, X. O. Extracellular RNA mediates and marks cancer progression. *Semin. Cancer Biol.* **28**, 14–23. 10.1016/j.semcancer.2014.04.010 (2014). 24783980 10.1016/j.semcancer.2014.04.010 PMC4162815

[16] Meldolesi, J. Exosomes and ectosomes in intercellular communication. *Curr. Biol. CB* **28**, R435–R444. 10.1016/j.cub.2018.01.059 (2018). 29689228 10.1016/j.cub.2018.01.059

[17] Mathieu, M., Martin-Jaular, L., Lavieu, G. & Théry, C. Specificities of secretion and uptake of exosomes and other extracellular vesicles for cell-to-cell communication. *Nat. Cell Biol.* **21**, 9–17. 10.1038/s41556-018-0250-9 (2019). 30602770 10.1038/s41556-018-0250-9

[18] Ratajczak, J. *et al.* Embryonic stem cell-derived microvesicles reprogram hematopoietic progenitors: Evidence for horizontal transfer of mRNA and protein delivery. *Leukemia* **20**, 847–856. 10.1038/sj.leu.2404132 (2006). 16453000 10.1038/sj.leu.2404132

[19] Valadi, H. *et al.* Exosome-mediated transfer of mRNAs and microRNAs is a novel mechanism of genetic exchange between cells. *Nat. Cell Biol.* **9**, 654–659. 10.1038/ncb1596 (2007). 17486113 10.1038/ncb1596

[20] Skog, J. *et al.* Glioblastoma microvesicles transport RNA and proteins that promote tumour growth and provide diagnostic biomarkers. *Nat. Cell Biol.* **10**, 1470–1476. 10.1038/ncb1800 (2008). 19011622 10.1038/ncb1800 PMC3423894

[21] Quesenberry, P. J., Aliotta, J., Deregibus, M. C. & Camussi, G. Role of extracellular RNA-carrying vesicles in cell differentiation and reprogramming. *Stem Cell Res. Ther.* **6**, 153. 10.1186/s13287-015-0150-x (2015). 26334526 10.1186/s13287-015-0150-x PMC4558901

[22] Fierabracci, A. *et al.* Recent advances in mesenchymal stem cell immunomodulation: the role of microvesicles. *Cell Transplant.* **24**, 133–149. 10.3727/096368913X675728 (2015). 24268069 10.3727/096368913X675728

[23] Ratajczak, M. Z. & Ratajczak, J. Horizontal transfer of RNA and proteins between cells by extracellular microvesicles: 14 years later. *Clin. Transl. Med.* **5**, 7. 10.1186/s40169-016-0087-4 (2016). 26943717 10.1186/s40169-016-0087-4 PMC4779088

[24] Phinney, D. G. & Pittenger, M. F. Concise review: MSC-derived exosomes for cell-free therapy. *Stem Cells (Dayton, Ohio)* **35**, 851–858. 10.1002/stem.2575 (2017). 28294454 10.1002/stem.2575

[25] Nikfarjam, S., Rezaie, J., Zolbanin, N. M. & Jafari, R. Mesenchymal stem cell derived-exosomes: A modern approach in translational medicine. *J. Transl. Med.* **18**, 449. 10.1186/s12967-020-02622-3 (2020). 33246476 10.1186/s12967-020-02622-3 PMC7691969

[26] Théry, C. *et al.* Minimal information for studies of extracellular vesicles 2018 (MISEV2018): A position statement of the International Society for Extracellular Vesicles and update of the MISEV2014 guidelines. *J. Extracell. Vesicles* **7**, 1535750. 10.1080/20013078.2018.1535750 (2018). 30637094 10.1080/20013078.2018.1535750 PMC6322352

[27] Tertel, T. *et al.* Imaging flow cytometry challenges the usefulness of classically used extracellular vesicle labeling dyes and qualifies the novel dye Exoria for the labeling of mesenchymal stromal cell-extracellular vesicle preparations. *Cytotherapy* **24**, 619–628. 10.1016/j.jcyt.2022.02.003 (2022). 35314115 10.1016/j.jcyt.2022.02.003

[28] Kloc, M., Bilinski, S., Dougherty, M. T., Brey, E. M. & Etkin, L. D. Formation, architecture and polarity of female germline cyst in Xenopus. *Dev. Biol.* **266**, 43–61. 10.1016/j.ydbio.2003.10.002 (2004). 14729477 10.1016/j.ydbio.2003.10.002

[29] Bilinski, S. M., Jaglarz, M. K., Dougherty, M. T. & Kloc, M. Electron microscopy, immunostaining, cytoskeleton visualization, in situ hybridization, and three-dimensional reconstruction of Xenopus oocytes. *Methods (San Diego, Calif.)* **51**, 11–19. 10.1016/j.ymeth.2009.12.003 (2010). 20018243 10.1016/j.ymeth.2009.12.003

[30] Fernández, O. *et al.* Research Group Study EudraCT 2008-004015-35. Adipose-derived mesenchymal stem cells (AdMSC) for the treatment of secondary-progressive multiple sclerosis: A triple blinded, placebo controlled, randomized phase I/II safety and feasibility study. *PloS One* **13**, e0195891. 10.1371/journal.pone.0195891 (2018). 29768414 10.1371/journal.pone.0195891 PMC5955528

[31] Makhlough, A. *et al.* Bone marrow-mesenchymal stromal cell infusion in patients with chronic kidney disease: A safety study with 18 months of follow-up. *Cytotherapy* **20**, 660–669. 10.1016/j.jcyt.2018.02.368 (2018). 29580865 10.1016/j.jcyt.2018.02.368

[32] Breitbach, M. *et al.* Potential risks of bone marrow cell transplantation into infarcted hearts. *Blood.* **110**, 1362–1369. 10.1182/blood-2006-12-063412 (2007). 17483296 10.1182/blood-2006-12-063412

[33] Guarro, M., Suñer, F., Lecina, M., Borrós, S. & Fornaguera, C. Efficient extracellular vesicles freeze-dry method for direct formulations preparation and use. *Colloids Surf. B Biointerfaces* **218**, 112745. 10.1016/j.colsurfb.2022.112745 (2022). 35930983 10.1016/j.colsurfb.2022.112745

[34] Wei, J. *et al.* Extracellular vesicle-mediated intercellular and interorgan crosstalk of pancreatic islet in health and diabetes. *Front. Endocrinol.* **14**, 1170237. 10.3389/fendo.2023.1170237 (2023). 10.3389/fendo.2023.1170237 PMC10248434 37305058

[35] Labusek, N. *et al.* Extracellular vesicles from immortalized mesenchymal stromal cells protect against neonatal hypoxic-ischemic brain injury. *Inflamm. Regen.* **43**, 24. 10.1186/s41232-023-00274-6 (2023). 37069694 10.1186/s41232-023-00274-6 PMC10108458

[36] Janockova, J., Slovinska, L., Harvanova, D., Spakova, T. & Rosocha, J. New therapeutic approaches of mesenchymal stem cells-derived exosomes. *J. Biomed. Sci.* **28**, 39. 10.1186/s12929-021-00736-4 (2021). 34030679 10.1186/s12929-021-00736-4 PMC8143902

[37] Lee, B. C., Kang, I. & Yu, K. R. Therapeutic features and updated clinical trials of mesenchymal stem cell (MSC)-derived exosomes. *J. Clin. Med.* **10**, 711. 10.3390/jcm10040711 (2021). 33670202 10.3390/jcm10040711 PMC7916919

[38] Hade, M. D., Suire, C. N. & Suo, Z. Mesenchymal stem cell-derived exosomes: Applications in regenerative medicine. *Cells* **10**, 1959. 10.3390/cells10081959 (2021). 34440728 10.3390/cells10081959 PMC8393426

[39] Ghoneim, M. A. *et al.* Transplantation of insulin-producing cells derived from human mesenchymal stromal/stem cells into diabetic humanized mice. *Stem Cell Res. Ther.* **13**, 350. 10.1186/s13287-022-03048-y (2022). 35883190 10.1186/s13287-022-03048-y PMC9327173

[40] Gardiner, C. *et al.* Techniques used for the isolation and characterization of extracellular vesicles: Results of a worldwide survey. *J. Extracell. Vesicles* **5**, 32945. 10.3402/jev.v5.32945 (2016). 27802845 10.3402/jev.v5.32945 PMC5090131

[41] Kornilov, R. *et al.* Efficient ultrafiltration-based protocol to deplete extracellular vesicles from fetal bovine serum. *J. Extracell. Vesicles* **7**, 1422674. 10.1080/20013078.2017.1422674 (2018). 29410778 10.1080/20013078.2017.1422674 PMC5795649

[42] Key parameters when using dynamic light scattering – 2018.azom.com.(n.d.) https://www.azom.com/article.aspx?ArticleID=16772.

[43] Rajagopal, J., Anderson, W. J., Kume, S., Martinez, O. I. & Melton, D. A. Insulin staining of ES cell progeny from insulin uptake. *Science (New York, N.Y.)* **299**, 363. 10.1126/science.1077838 (2003). 12532008 10.1126/science.1077838

[44] Gabr, M. M. *et al.* Differentiation of human bone marrow-derived mesenchymal stem cells into insulin-producing cells: Evidence for further maturation in vivo. *Biomed. Res. Int.* **2015**, 575837. 10.1155/2015/575837 (2015). 26064925 10.1155/2015/575837 PMC4443784

[45] Favaro, E. *et al.* Human mesenchymal stem cells and derived extracellular vesicles induce regulatory dendritic cells in type 1 diabetic patients. *Diabetologia* **59**, 325–333. 10.1007/s00125-015-3808-0 (2016). 26592240 10.1007/s00125-015-3808-0

[46] Shigemoto-Kuroda, T. *et al.* MSC-derived extracellular vesicles attenuate immune responses in two autoimmune murine models: Type 1 diabetes and uveoretinitis. *Stem Cell Rep.* **8**, 1214–1225. 10.1016/j.stemcr.2017.04.008 (2017). 10.1016/j.stemcr.2017.04.008 PMC5425726 28494937

[47] Song, J. *et al.* Mesenchymal stromal cells ameliorate diabetes-induced muscle atrophy through exosomes by enhancing AMPK/ULK1-mediated autophagy. *J. Cachexia Sarcopenia Muscle.* **14**, 915–929. 10.1002/jcsm.13177 (2023). 36708027 10.1002/jcsm.13177 PMC10067482

[48] Sharma, R. *et al.* Exosomes secreted by umbilical cord blood-derived mesenchymal stem cell attenuate diabetes in mice. *J. Diabetes Res.* **2021**, 9534574. 10.1155/2021/9534574 (2021). 34926699 10.1155/2021/9534574 PMC8683199

[49] Sun, Y. *et al.* Human mesenchymal stem cell derived exosomes alleviate type 2 diabetes mellitus by reversing peripheral insulin resistance and relieving β-cell destruction. *ACS Nano* **12**, 7613–7628. 10.1021/acsnano.7b07643 (2018). 30052036 10.1021/acsnano.7b07643

[50] Kulaj, K. *et al.* Adipocyte-derived extracellular vesicles increase insulin secretion through transport of insulinotropic protein cargo. *Nat. Commun.* **14**, 709. 10.1038/s41467-023-36148-1 (2023). 36759608 10.1038/s41467-023-36148-1 PMC9911726

[51] Sun, Y., Mao, Q., Shen, C., Wang, C. & Jia, W. Exosomes from β-cells alleviated hyperglycemia and enhanced angiogenesis in islets of streptozotocin-induced diabetic mice. *Diabetes Metab. Syndr. Obes.* **12**, 2053–2064. 10.2147/DMSO.S213400 (2019). 31632115 10.2147/DMSO.S213400 PMC6790122

[52] Guo, Q. *et al.* Exosomes from β-cells promote differentiation of induced pluripotent stem cells into insulin-producing cells through microRNA-dependent mechanisms. *Diabetes Metab. Syndr. Obes.* **14**, 4767–4782. 10.2147/DMSO.S342647 (2021). 34934332 10.2147/DMSO.S342647 PMC8678630

[53] Bai, C. *et al.* miR-212/132-enriched extracellular vesicles promote differentiation of induced pluripotent stem cells into pancreatic beta cells. *Front. Cell Dev. Biol.* **9**, 673231. 10.3389/fcell.2021.673231 (2021). 34055806 10.3389/fcell.2021.673231 PMC8155495

[54] Mandal, P., De, D., Im, D. U., Um, S. H. & Kim, K. K. Exosome-mediated differentiation of mouse embryonic fibroblasts and exocrine cells into β-like cells and the identification of key miRNAs for differentiation. *Biomedicines* **8**, 485. 10.3390/biomedicines8110485 (2020). 33182285 10.3390/biomedicines8110485 PMC7695333

[55] Cheng, K. *et al.* High passage MIN6 cells have impaired insulin secretion with impaired glucose and lipid oxidation. *PloS One* **7**, e40868. 10.1371/journal.pone.0040868 (2012). 22808281 10.1371/journal.pone.0040868 PMC3396628

[56] Henquin, J. C., Nenquin, M., Guiot, Y., Rahier, J. & Sempoux, C. Human insulinomas show distinct patterns of insulin secretion in vitro. *Diabetes* **64**, 3543–3553. 10.2337/db15-0527 (2015). 26116696 10.2337/db15-0527

[57] Deregibus, M. C. *et al.* Endothelial progenitor cell derived microvesicles activate an angiogenic program in endothelial cells by a horizontal transfer of mRNA. *Blood* **110**, 2440–2448. 10.1182/blood-2007-03-078709 (2007). 17536014 10.1182/blood-2007-03-078709

[58] Herrera, M. B. *et al.* Human liver stem cell-derived microvesicles accelerate hepatic regeneration in hepatectomized rats. *J. Cell Mol. Med.* **14**, 1605–1618. 10.1111/j.1582-4934.2009.00860.x (2010). 19650833 10.1111/j.1582-4934.2009.00860.x PMC3060338

[59] Aliotta, J. M. *et al.* Microvesicle entry into marrow cells mediates tissue-specific changes in mRNA by direct delivery of mRNA and induction of transcription. *Exp. Hematol.* **38**, 233–245. 10.1016/j.exphem.2010.01.002 (2010). 20079801 10.1016/j.exphem.2010.01.002 PMC2829939

[60] Yuan, A. *et al.* Transfer of microRNAs by embryonic stem cell microvesicles. *PloS One* **4**, e4722. 10.1371/journal.pone.0004722 (2009). 19266099 10.1371/journal.pone.0004722 PMC2648987

[61] Chen, X., Liang, H., Zhang, J., Zen, K. & Zhang, C. Y. Secreted microRNAs: A new form of intercellular communication. *Trends. Cell Biol.* **22**, 125–132. 10.1016/j.tcb.2011.12.001 (2012). 22260888 10.1016/j.tcb.2011.12.001

[62] Collino, F. *et al.* Microvesicles derived from adult human bone marrow and tissue specific mesenchymal stem cells shuttle selected pattern of miRNAs. *PloS One* **5**, e11803. 10.1371/journal.pone.0011803 (2010). 20668554 10.1371/journal.pone.0011803 PMC2910725

[63] Camussi, G. *et al.* Exosome/microvesicle-mediated epigenetic reprogramming of cells. *Am. J. Cancer Res.* **1**, 98–110 (2011). 21969178 PMC3180104

[64] Toh, W. S., Lai, R. C., Zhang, B. & Lim, S. K. MSC exosome works through a protein-based mechanism of action. *Biochem. Soc. Trans.* **46**, 843–853. 10.1042/BST20180079 (2018). 29986939 10.1042/BST20180079 PMC6103455

[65] Lai, R. C. *et al.* Proteolytic potential of the MSC exosome proteome: implications for an exosome-mediated delivery of therapeutic proteasome. *Int. J. Proteomics* **2012**, 971907. 10.1155/2012/971907 (2012). 22852084 10.1155/2012/971907 PMC3407643

