# Supplementary material for: Modulation of naïve mesenchymal stromal cells by extracellular vesicles derived from insulin-producing cells: an in vitro study
Source: Sci Rep. 2024 Aug 1;14:17844. doi: 10.1038/s41598-024-68104-4 (PMC11294623; doi:10.1038/s41598-024-68104-4)

**Additional file 1: Data S1**

**Transmission electron microscopy:**

Freshly isolated EVs suspension was applied to a copper mesh Formvar coated carbon-stabilized grid and allowed to adsorb for 5 min. The preparation was then allowed to dry completely. Images were obtained with a transmission electron microscope (JEOL, Japan).

**Additional file 1: Data S2**

**Particle size distribution of EVs:**

Particle size distribution was performed using Nano-ZS90 (Malvern, UK). EVs suspensions were diluted with purified water to a concentration of 300 µg/mL. The viscosity of the dispersant was set as 0.8872 cP and its refractive index (IR) was 1.33 at 25⁰C.

**Additional file 1: Data S3**

**Characterization of specific EVs protein by flow cytometry:**

1. **Detection of CD63+ EVs:** after obtaining EVs by differential ultracentrifugation protocol, 50 μL of the superparamagnetic capture beads CD 63 [EVs isolation and analysis Kit- Flow Cytometry, cell culture (CD63/ CD9)] (# ab267478, Abcam Cambridge, UK) were added to 15 µg of isolated **EVs** in cytometer tube. The sample (bead-bound **EVs**) were gently mixed by pipetting several times, then incubated in the dark overnight at room temperature. The sample was then washed with 1 ml of Assay Buffer 1X (# ab267478). The cytometer tubes were put on a magnetic rack (Stem cell Technologies Inc 18000) for 5 minutes, then supernatant was removed by hand-decanting to collect bead-bound **EV.**
2. **Detection of CD 9+ EVs:** Anti-CD9 antibody PE [clone VJ1/20] (# ab267478) (0.5 µg/test) was added to the tube containing the bead-bound EVs. The sample was gently mixed by pipetting then incubated in the dark for 60 minutes at 2-8ºC. The sample was washed with 1 ml of Assay Buffer 1X, and bead-bound **EVs** were collected as previously described.
3. **Quantitation by flowcytometry:** The sample was resuspended in 350 μL Assay Buffer and the labeled beads were identified using blue & red laser with a wavelength of 488nm & 633nm simultaneously by the FACS ARIA III cell sorter (Becton, Dickinson). A total of 10,000 events were obtained. Unstained cells served as negative controls. The data were analyzed by FACS DIVA software (Becton, Dickinson).

**Additional file 1: Data S4**

**Western blotting:**

EVs were suspended in 100 µL PBS. Protein concentration was quantified by BCA protein assay kit (EMD Millipore, MA, USA). Each sample was mixed with an equal volume of 2X Laemmli buffer and heated at 95⁰C for 5 minutes. Samples were then separated by gel electrophoresis (25 μg/well) and transferred to PVDF membrane using Trans-Blot Turbo RTA Transfer kit and Trans-Blot Turbo Transfer System (BIO-RAD, CA, USA). The membrane was blocked with Every Blot blocking buffer (BIO-RAD) with shaking for 1 h at RT. Then, the membranes were individually incubated with a primary antibody: mouse anti human CD81 (1:1000), mouse anti human CD9 (1:1000) (BIO-RAD), mouse anti human CD63 (1:1000) (Abcam, UK), Rabbit anti-syntenin (1:1000) (abcam), Rabbit anti-TSG101 (1:1000) (SIGMA-ALDRICH), goat anti-human GRP94 (1:1000) (BIO-RAD), rabbit anti-Calnexin (1:2000) (abcam), and rabbit anti-CD105 (1:1000) (abcam) overnight at 4⁰C with continuous shaking. Next day, the membranes were washed 3 times with 1X TBS-T (0.1 % Tween 20) and then incubated with the secondary antibodies: goat anti mouse IgG1:HRP (1:10000), goat anti mouse IgG2a:HRP (1:1000), and goat anti rabbit IgG (H+L):HRP (1:1000) (BIO-RAD) with Precision protein Strep Tactin-HRP conjugate (1:15000) (BIO-RAD) at 4⁰C for 1.5 h on a shaker. After washing the membrane 3 times with 1X TBS-T and adding ClarityTM Western ECL substrate (BIO-RAD), the Blots were imaged using the ChemiDocTM XRS+ system (BIO-RAD).

| **Additional file 1: Data S5**  **Proportion of hormone-positive cells under different experimental condition** | **EVs/ml/total cells** | **Duration of**  **co-culture** | **Naïve MSCs with educated exosomes** | |
| --- | --- | --- | --- | --- |
|  |  |  | **Insulin %** | **C-peptide %** |
| **Exp 1** | **10µg/ml/1x10^5^** | **24 hours** | **7.6** | **5.5** |
| **Exp 2** | **20µg/ml/1x10^5^** | **24 hours** | **1.09** | **0.73** |
| **Exp3** | **40µg/ml/1x10^5^** | **24 hours** | **8.56** | **6.79** |
| **Exp 4** | **80µg/ml/1x10^5^** | **24 hours** | **11.2** | **8.91** |
| **Exp 5** | **80µg/ml/1x10^5^** | **24 hours** | **12.2** | **9.8** |
| **Exp 6** | **80µg/ml/1x10^5^** | **24 hours** | **19.4** | **18.2** |
|  |  | **72 hours** | **16.3** | **14.4** |
|  |  | **Every 3 days** | **13** | **11.2** |
|  | **EVs/ml/total cells** | **Duration of**  **co-culture** | **Naïve MSCs with educated exosomes** | |
|  |  |  | **Insulin %** | **C-peptide %** |
| **Exp 7** | **80µg/ml/x10^5^** | **24 hours** | **15.4** | **14.5** |
|  | **120µg/ml/1x10^5^** |  | **13.2** | **11.7** |
|  | **160µg/ml/1x10^5^** |  | **16** | **15** |
|  | **200µg/ml/1x10^5^** |  | **11.6** | **9.32** |
| **Exp 8** | **80µg/ml/1x10^5^** | **24 hours** | **16.9** | **14** |
|  |  | **Every 3 days** | **15.7** | **13.8** |
| **Exp 9** | **80µg/ml/1x10^5^** | **24 hours** | **18.2** | **16.3** |
| **Exp 10** | **80µg/ml/1x10^5^** | **24 hours** | **15.9** | **13.4** |

**Proportion of hormone positive cells under different experimental condition**

**Additional file 1: Data S6**

**Quantification of insulin and c-peptide positive cells by flow cytometry:**

At the end of differentiation 1×106 cells were collected in αMEM (alpha minimum essential media) (Sigma Aldrich St. Louis, MO, USA) containing 10µl of Brefeldin A (Sigma) and incubated at 37˚c in a 5% CO2 incubator for 4 hours. The cell preparation was centrifuged at 1200 RPM for 10 minutes, the supernatant was discarded, and the cell pellet was re-suspended in 2ml of Phosphate Buffer Saline (PBS) (Sigma) followed by centrifugation at 1200 RPM for 10 min at 4°C. The cells were than fixed by incubation with 4% paraformaldehyde solution (Thermo Fisher Scientific, Waltham, US) in a concentration of 1 ml/106 cells for 30 min on ice in a dark place. Thereafter, the cells were washed by 2ml PBS once & centrifugated at 1200 RPM at for 10 min at 4°C. Permeabilization was carried out by intracellular permeabilization solution [Tritone X (Thermo Fisher) + Fetal Bovine Serum (Hyclon, Logan, UT, USA) + PBS (Sigma)] in a concentration of 1ml/106 cells and incubated for 30 min on ice in a dark place. The cells were washed again with intracellular permeabilization solution and centrifuged at 1200 RPM for 10 min at 4°C. The supernatant was discarded, and 10 µL of diluted primary monoclonal antibodies (1:200) for insulin and c-peptide were added to the cell pellet and incubated overnight at 4°c. Cells were washed by 2 ml of intracellular permeabilization solution and centrifuged at 1200 RPM for 10 min at 4°C and the supernatant was discarded. Ten µL of diluted Secondary antibody (1:1000) were added to the cell pellet for 30-60 min on ice in a dark place. The cells were then washed by 500 µL stain buffer (Becton, Dickinson, San Jose, CA, USA) once, centrifuged at 1200 RPM for 10 min at 4°C and the supernatant was discarded. Finally, the cells were re-suspended in 500 µL stain buffer.

**Additional file 1: Data S7**

**The Phenotype of MSCs**

|  | **CD 14** | **CD 34** | **CD 45** | **CD 73** | **CD 90** | **CD 105** |
| --- | --- | --- | --- | --- | --- | --- |
| **Donor 1** | 0.7  1.2  1.8 | 1.1  0.3  1.2 | 0.5  0.1  0.2 | 98.9  99.6  92.0 | 98.1  99.4  99.5 | 99.9  96.7  94.0 |
| **Donor 2** | 1.9  2.4  2.6 | 2.2  1.6  2.1 | 0.1  0.3  0.4 | 91.4  93.8  99.2 | 98.8  99.3  99.5 | 97.1  97.8  93.0 |
| **Donor 3** | 1.4  0.8  1.3 | 1.5  1.4  1.9 | 0.1  0.05  0.1 | 97.1  92.0  92.0 | 99.5  92.6  96.9 | 95.8  95.2  97.7 |

**Additional File 1: Data S8**

**
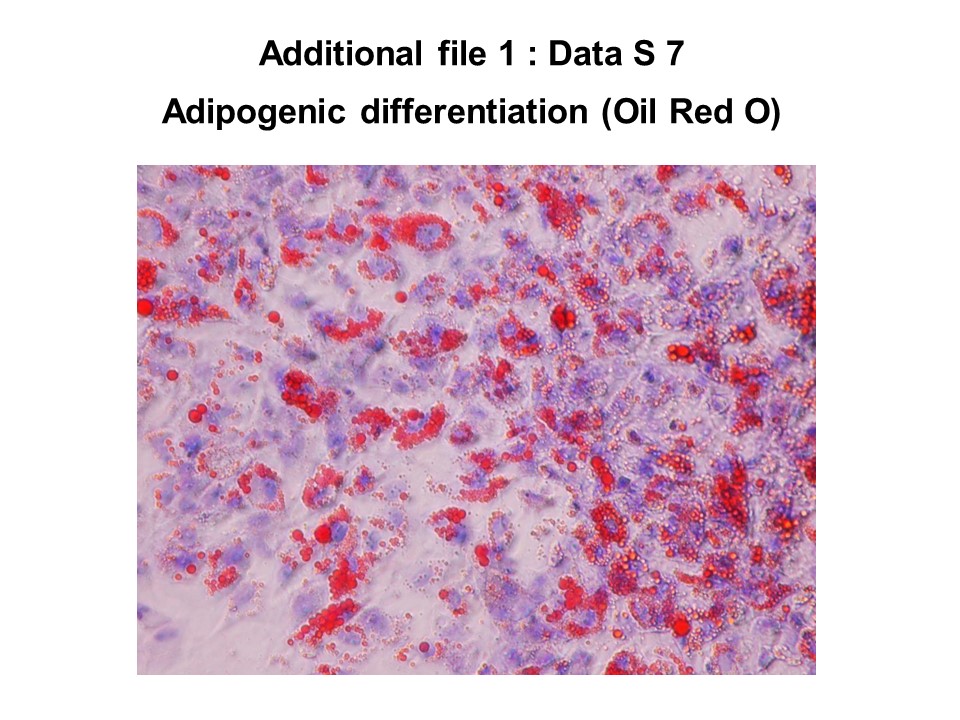
**

**
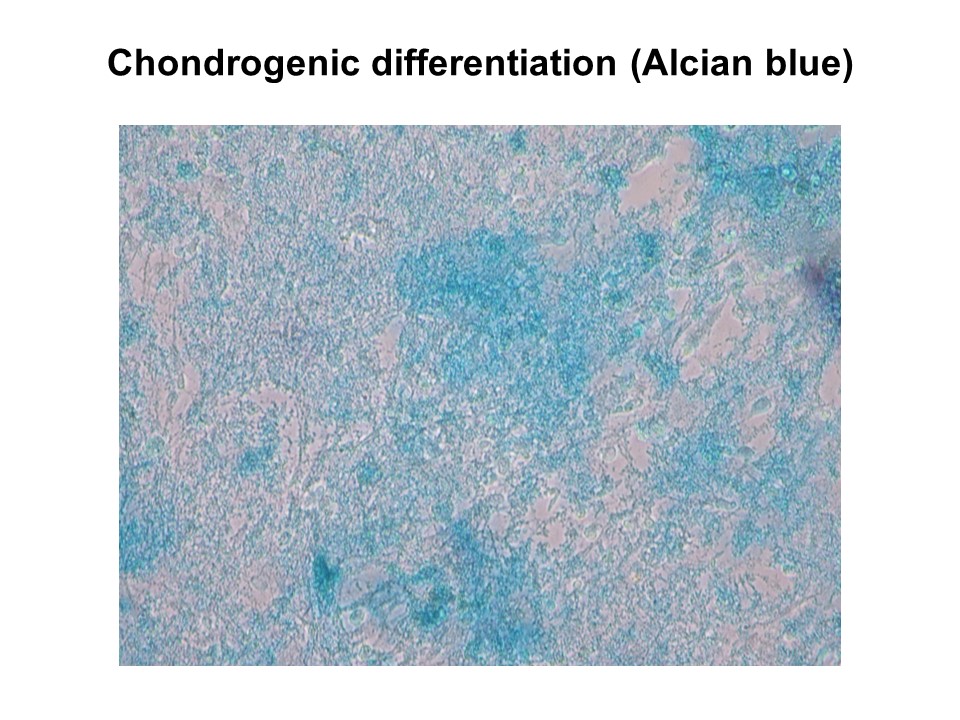
**


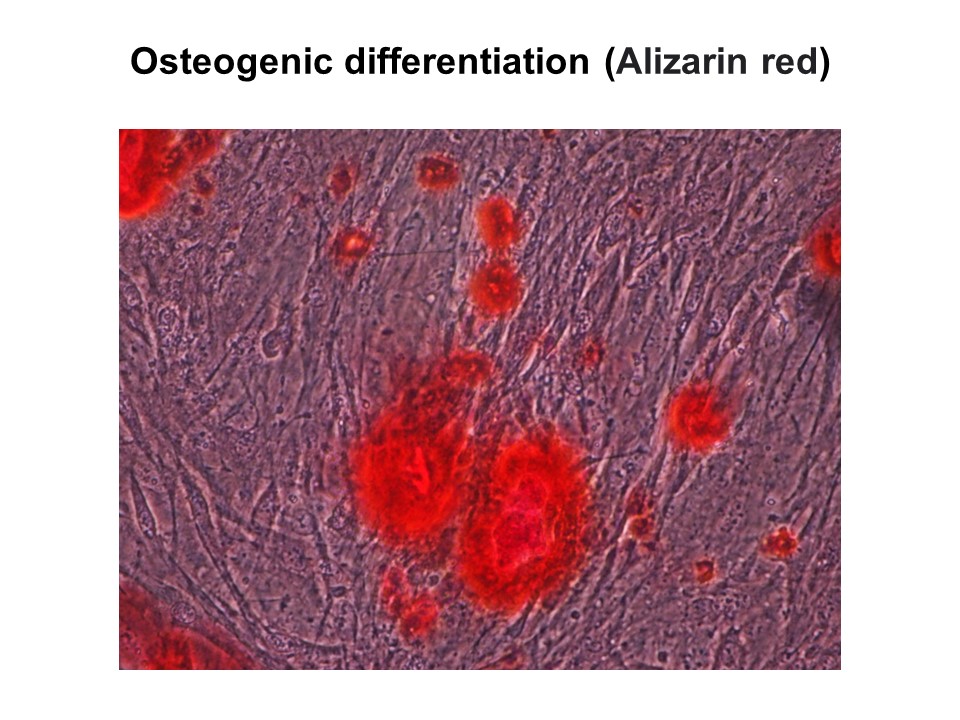


**Additional file 1: Data S9**

**Proportion of hormone-positive cells**

|  | **Doner Cells** | | | | **Recipients Cells** | | | | | |
| --- | --- | --- | --- | --- | --- | --- | --- | --- | --- | --- |
|  | **Native MSCs** | | **Differentiated cells** | | **Native MSCs**  **+ educated EVs** | | **Native MSCs**  **+ uneducated EVs** | | **HG media only** | |
|  | **Insulin**  **(%)** | **C-peptide**  **(%)** | **Insulin**  **(%)** | **C-peptide**  **(%)** | **Insulin**  **(%)** | **C-peptide**  **(%)** | **Insulin**  **(%)** | **C-peptide**  **(%)** | **Insulin**  **(%)** | **C-peptide**  **(%)** |
| **Exp 1** | **4.7** | **3.4** | **29.6** | **20.4** | **11.2** | **8.91** | **3.21** | **1.1** | **---** | **---** |
| **Exp 2** | **0.89** | **0.43** | **21** | **14.9** | **12.2** | **9.8** | **6.55** | **5.82** | **---** | **---** |
| **Exp 3** | **0.73** | **0.56** | **21.4** | **14.2** | **19.4** | **18.2** | **---** | **--** | **1.19** | **0.87** |
| **Exp 4** | **0.94** | **0.77** | **28.3** | **21.4** | **15.4** | **14.5** | **----** | **---** | **1.38** | **1.07** |
| **Exp 5** | **0.83** | **0.76** | **19.4** | **18.2** | **16.9** | **14** | **0.76** | **0.45** | **---** | **---** |
| **Exp 6** | **0.83** | **0.76** | **19.4** | **18.2** | **18.2** | **16.3** | **0.89** | **0.43** | **---** | **---** |
| **Median**  **Min.**  **Max.** | **0.86** | **0.76** | **21.2** | **18.2** | **16.15** | **14.25** | **2.05** | **0.77** | **1.28** | **0.97** |
|  | **0.73** | **0.43** | **19.4** | **14.2** | **11.2** | **8.91** | **0.76** | **0.43** | **1.19** | **0.87** |
|  | **4.7** | **3.4** | **29.6** | **21.4** | **19.4** | **18.2** | **6.55** | **5.82** | **1.38** | **1.07** |

**Additional file 1: Data S10**

**Relative Gene expression results, (Mean values of 8 study)**

| **Genes** | **Donor  (AT-MSCs)** | **Undifferentiated MSCs** | **MSCs differentiated by the Conventional protocol** | **MSCs co-cultured with Educated EVs** | **MSCs co-cultured with Uneducated EVs** |
| --- | --- | --- | --- | --- | --- |
| **INS** | **Donor 1** | **1** | **-** | **1.3** | **1.05** |
|  | **Donor 1** | **1** | **-** | **1.71** | **0.74** |
|  | **Donor 1** | **1** | **4.55** | **1.62** | **0.54** |
|  | **Donor 2** | **1** | **1.34** | **1.21** | **0.86** |
|  | **Donor 2** | **1** | **2.2** | **1.1** | **-** |
|  | **Donor 2** | **1** | **5.06** | **-** | **-** |
|  | **Donor 3** | **1** | **-** | **1.5** | **1.19** |
|  | **Donor 3** | **1** | **11.88** | **4.32** | **2.54** |
|  | **Median (min, max)** |  | **4.5 (1.34,11.88)** | **1.5 (1.1,4.32)** | **0.955 (0.54,2.54)** |
|  |  | | | | |
| **GCG** | **Donor 1** | **1** | **-** | **0.71** | **0.85** |
|  | **Donor 1** | **1** | **-** | **1.74** | **0.77** |
|  | **Donor 1** | **1** | **4.29** | **1.54** | **3.26** |
|  | **Donor 2** | **1** | **1.52** | **1.16** | **1.06** |
|  | **Donor 2** | **1** | **2.3** | **1.0** | **-** |
|  | **Donor 2** | **1** | **5.39** | **-** | **-** |
|  | **Donor 3** | **1** | **-** | **1.13** | **0.62** |
|  | **Donor 3** | **1** | **5.26** | **4.6** | **1.62** |
|  | **Median (min, max)** |  | **4.2 (1.52,5.39)** | **1.16 (0.71,4.6)** | **0.955 (0.62,3.26)** |
|  |  | | | | |
| **SST** | **Donor 1** | **1** | **-** | **0.55** | **1.11** |
|  | **Donor 1** | **1** | **-** | **0.9** | **0.32** |
|  | **Donor 1** | **1** | **12.82** | **4.0** | **8.41** |
|  | **Donor 2** | **1** | **4.36** | **2.04** | **3.88** |
|  | **Donor 2** | **1** | **3.13** | **3.1** | **-** |
|  | **Donor 2** | **1** | **50.94** | **-** | **-** |
|  | **Donor 3** | **1** | **-** | **1.52** | **0.54** |
|  | **Donor 3** | **1** | **13.87** | **7.02** | **4.51** |
|  | **Median (min, max)** |  | **12.82 (3.13,50.94)** | **2.04 (0.55,7.02)** | **2.49 (0.32,8.41)** |

| **PDX-1** | **Donor 1** | **1** | **-** | | **1.0** | | **0.97** | |
| --- | --- | --- | --- | --- | --- | --- | --- | --- |
|  | **Donor 1** | **1** | **-** | | **1.89** | | **0.83** | |
|  | **Donor 1** | **1** | **5.9** | | **1.62** | | **3.3** | |
|  | **Donor 2** | **1** | **1.67** | | **1.04** | | **0.74** | |
|  | **Donor 2** | **1** | **2.1** | | **1.3** | | **-** | |
|  | **Donor 2** | **1** | **5.21** | | **-** | | **-** | |
|  | **Donor 3** | **1** | **-** | | **1.1** | | **0.7** | |
|  | **Donor 3** | **1** | **9.42** | | **3.22** | | **1.49** | |
|  | **Median (min,max)** |  | **5.21 (1.67,9.42)** | | **1.3 (1,3.22)** | | **0.9 (0.7,3.3)** | |
|  |  | | | | | | | |
| **NES** | **Donor 1** | **1** | **-** | | **0.86** | | **0.89** | |
|  | **Donor 1** | **1** | **-** | | **1.25** | | **1.01** | |
|  | **Donor 1** | **1** | **4.8** | | **1.5** | | **0.71** | |
|  | **Donor 2** | **1** | **1.11** | | **0.83** | | **0.35** | |
|  | **Donor 2** | **1** | **0.52** | | **0.87** | | **-** | |
|  | **Donor 2** | **1** | **5.86** | | **-** | | **-** | |
|  | **Donor 3** | **1** | **-** | | **0.93** | | **0.62** | |
|  | **Donor 3** | **1** | **8.14** | | **3.56** | | **1.57** | |
|  | **Median (min,max)** |  | **4.8 (0.52,8.14)** | | **0.93 (0.83,3.56)** | | **0.8 (0.35,1.57)** | |
|  |  | | | | | | | |
| **MAFA** | **Donor 1** | **1** | **-** | | **1.4** | | **0.88** | |
|  | **Donor 1** | **1** | **-** | | **1.32** | | **0.88** | |
|  | **Donor 1** | **1** | **3.93** | | **1.17** | | **0.46** | |
|  | **Donor 2** | **1** | **1.11** | | **0.48** | | **0.31** | |
|  | **Donor 2** | **1** | **0.5** | | **0.49** | | **-** | |
|  | **Donor 2** | **1** | **5.07** | | **-** | | **-** | |
|  | **Donor 3** | **1** | **-** | | **0.90** | | **0.56** | |
|  | **Donor 3** | **1** | **10.98** | | **3.99** | | **2.74** | |
|  | **Median (min,max)** |  | **3.9 (0.5,1.98)** | | **1.17 (0.48,3.99)** | | **0.72 (0.31,2.74)** | |
|  |  | | | | | | | |
| **MAFB** | **Donor 1** | **1** | **-** | | **2.0** | **2.09** | | |
|  | **Donor 1** | **1** | **-** | | **0.36** | **2.08** | | |
|  | **Donor 1** | **1** | **17.16** | | **4.00** | **9.32** | | |
|  | **Donor 2** | **1** | **0.5** | | **0.35** | **0.43** | | |
|  | **Donor 2** | **1** | **3.02** | | **6.22** | **-** | | |
|  | **Donor 2** | **1** | **10.82** | | **-** | **-** | | |
|  | **Donor 3** | **1** | **-** | | **0.61** | **0.54** | | |
|  | **Donor 3** | **1** | **2.65** | | **2.69** | **0.54** | | |
|  | **Median (min,max)** |  | **3.02 (0.5,17.16)** | | **2 (0.35,6.22)** | **1.31 (0.43,9.32)** | | |
| **GCK** | **Donor 1** | **1** | | **-** | **0.87** | | | **0.76** |
|  | **Donor 1** | **1** | | **-** | **1.21** | | | **0.77** |
|  | **Donor 1** | **1** | | **6.15** | **1.90** | | | **1.97** |
|  | **Donor 2** | **1** | | **1.08** | **0.66** | | | **0.31** |
|  | **Donor 2** | **1** | | **0.22** | **0.47** | | | **-** |
|  | **Donor 2** | **1** | | **5.34** | **-** | | | **-** |
|  | **Donor 3** | **1** | | **-** | **0.88** | | | **0.75** |
|  | **Donor 3** | **1** | | **9.38** | **3.80** | | | **1.32** |
|  | **Median (min,max)** |  | | **5.3 (0.22,9.38)** | **0.88 (0.47,3.8)** | | | **0.76 (0.31,1.97)** |
|  |  | | | | | | | |
| **Glut-2** | **Donor 1** | **1** | | **-** | **1.05** | | **1.02** | |
|  | **Donor 1** | **1** | | **-** | **1.49** | | **0.86** | |
|  | **Donor 1** | **1** | | **4.42** | **1.39** | | **0.40** | |
|  | **Donor 2** | **1** | | **1.42** | **0.84** | | **0.12** | |
|  | **Donor 2** | **1** | | **0.49** | **0.52** | | **-** | |
|  | **Donor 2** | **1** | | **5.23** | **-** | | **-** | |
|  | **Donor 3** | **1** | | **-** | **1.06** | | **0.63** | |
|  | **Donor 3** | **1** | | **8.64** | **4.14** | | **1.86** | |
|  | **Median (min,max)** |  | | **4.42 (0.49,8.64)** | **1.06 (0.52,4.14)** | | **0.74 (0.12,1.86)** | |
|  |  | | | | | | | |
| **NEUROD1** | **Donor 1** | **1** | | **-** | **1.12** | | **1.09** | |
|  | **Donor 1** | **1** | | **-** | **1.22** | | **0.79** | |
|  | **Donor 1** | **1** | | **5.58** | **1.53** | | **0.61** | |
|  | **Donor 2** | **1** | | **1.27** | **0.74** | | **0.16** | |
|  | **Donor 2** | **1** | | **0.82** | **0.45** | | **-** | |
|  | **Donor 2** | **1** | | **6.63** | **-** | | **-** | |
|  | **Donor 3** | **1** | | **-** | **1.00** | | **0.78** | |
|  | **Donor 3** | **1** | | **9.10** | **3.59** | | **1.51** | |
|  | **Median (min,max)** |  | | **5.58 (0.82,9.1)** | **1.12 (0.45,3.59)** | | **0.78 (0.16,1.51)** | |
|  | | | | | | | | |
| **RFX6** | **Donor 1** | **1** | | **-** | **0.93** | | **0.90** | |
|  | **Donor 1** | **1** | | **-** | **1.37** | | **0.74** | |
|  | **Donor 1** | **1** | | **5.50** | **1.45** | | **0.55** | |
|  | **Donor 2** | **1** | | **1.32** | **0.86** | | **0.12** | |
|  | **Donor 2** | **1** | | **0.5** | **0.36** | | **-** | |
|  | **Donor 2** | **1** | | **5.67** | **-** | | **-** | |
|  | **Donor 3** | **1** | | **-** | **1.24** | | **0.67** | |
|  | **Donor 3** | **1** | | **6.15** | **4.28** | | **1.46** | |
|  | **Median (min,max)** |  | | **5.5 (0.5,6.15)** | **1.24 (0.36,4.28)** | | **0.705 (0.12,1.46)** | |

| **NGN3** | **Donor 1** | **1** | **-** | **1.04** | **0.91** |
| --- | --- | --- | --- | --- | --- |
|  | **Donor 1** | **1** | **-** | **1.50** | **0.74** |
|  | **Donor 1** | **1** | **11.21** | **2.54** | **1.43** |
|  | **Donor 2** | **1** | **1.53** | **0.75** | **0.32** |
|  | **Donor 2** | **1** | **0.49** | **0.23** | **-** |
|  | **Donor 2** | **1** | **6.52** | **-** | **-** |
|  | **Donor 3** | **1** | **-** | **1.55** | **1.26** |
|  | **Donor 3** | **1** | **11.34** | **4.09** | **2.11** |
|  | **Median (min,max)** |  | **6.5 (0.49,11.34)** | **1.5 (0.23,4.09)** | **1.08 (0.32,2.11)** |
|  |  | | | | |
| **PAX4** | **Donor 1** | **1** | **-** | **0.38** | **0.48** |
|  | **Donor 1** | **1** | **-** | **1.25** | **0.83** |
|  | **Donor 1** | **1** | **53.15** | **29.47** | **16.33** |
|  | **Donor 2** | **1** | **1.00** | **1.37** | **0.50** |
|  | **Donor 2** | **1** | **0.59** | **0.45** | **-** |
|  | **Donor 2** | **1** | **5.75** | **-** | **-** |
|  | **Donor 3** | **1** | **-** | **1.43** | **1.46** |
|  | **Donor 3** | **1** | **5.32** | **3.21** | **1.63** |
|  | **Median (min,max)** |  | **5.32 (0.59,53.15)** | **1.37 (0.38,29.47)** | **1.14 (0.48,16.33)** |
|  |  | | | | |
| **ERRɣ** | **Donor 1** | **1** | **-** | **1.10** | **1.03** |
|  | **Donor 1** | **1** | **-** | **1.52** | **0.79** |
|  | **Donor 1** | **1** | **5.80** | **1.71** | **0.49** |
|  | **Donor 2** | **1** | **1.31** | **0.74** | **0.15** |
|  | **Donor 2** | **1** | **0.85** | **0.54** | **-** |
|  | **Donor 2** | **1** | **4.53** | **-** | **-** |
|  | **Donor 3** | **1** | **-** | **1.44** | **1.12** |
|  | **Donor 3** | **1** | **6.29** | **3.86** | **1.96** |
|  | **Median (min,max)** |  | **4.53 (0.85,6.29)** | **1.44 (0.54,3.86)** | **0.91 (0.15,1.96)** |

**Additional file 1: Data S11**

**Human insulin release in response to glucose challenge**

**Conventional protocol**

|  | **Human insulin release (ng/μg protein/ hr)** | | |
| --- | --- | --- | --- |
|  | **5.5 mM** | **12 mM** | **25 mM** |
|  | **1.8** | **2.3** | **3.8** |
|  | **2.2** | **2.7** | **6.5** |
|  | **2.5** | **2.9** | **6.8** |
|  | **2.6** | **3.1** | **7** |
|  | **2.3** | **2.8** | **6.7** |
|  | **2.5** | **3** | **6.9** |
| **Median  (Min , Max)** | **2.4**  **(1.8 , 2.6)** | **2.85 (2.3 , 3.1)** | **6.75 (3.8 , 7)** |

**Human insulin release in response to glucose challenge**

**MSCs co-cultured with educated exosomes**

|  | **Human insulin release (ng/μg protein/ hr)** | | |
| --- | --- | --- | --- |
|  | **5.5 mM** | **12 mM** | **25 mM** |
|  | **1.2** | **1.6** | **2.0** |
|  | **1.5** | **1.8** | **2.4** |
|  | **1.4** | **1.9** | **2.5** |
|  | **1.2** | **1.6** | **2.3** |
|  | **1.3** | **1.8** | **2.4** |
|  | **1.5** | **2** | **2.6** |
| **Median  (Min , Max)** | **1.35**  **(1.2 , 1.5)** | **1.8 (1.6 , 2)** | **2.4 (2.0 , 2.6)** |

**Human insulin release in response to glucose challenge**

**MSCs co-cultured with uneducated exosomes**

|  | **Human insulin release (ng/μg protein/ hr)** | | |
| --- | --- | --- | --- |
|  | **5.5 mM** | **12 mM** | **25 mM** |
|  | **0.008** | **0.016** | **0.02** |
|  | **0.007** | **0.013** | **0.018** |
|  | **0.006** | **0.012** | **0.018** |
|  | **0.003** | **0.010** | **0.016** |
|  | **0.005** | **0.013** | **0.02** |
|  | **0.004** | **0.011** | **0.017** |
| **Median  (Min , Max)** | **0.0055**  **(0.003 , 0.008)** | **0.0125 (0.01 , 0.016)** | **0.018 (0.016 , 0.02)** |

**Human C-peptide release in response to glucose challenge**

**Conventional protocol**

|  | **Human C-peptide release (ng/μg protein/ hr)** | | |
| --- | --- | --- | --- |
|  | **5.5 mM** | **12 mM** | **25 mM** |
|  | **1.1** | **1.8** | **3.2** |
|  | **1.8** | **2.2** | **4.5** |
|  | **2.1** | **2.3** | **4.7** |
|  | **2.2** | **2.7** | **5** |
|  | **1.9** | **2.1** | **4.9** |
|  | **2** | **2.2** | **5.2** |
| **Median  (Min , Max)** | **1.95**  **(1.1 , 2.2)** | **2.2 (1.8 , 2.7)** | **4.8 (3.2 , 5.2)** |

**Human C-peptide release in response to glucose challenge**

**MSCs co-cultured with educated exosomes**

|  | **Human C-peptide release (ng/μg protein/ hr)** | | |
| --- | --- | --- | --- |
|  | **5.5 mM** | **12 mM** | **25 mM** |
|  | **0.8** | **1.2** | **1.6** |
|  | **1.1** | **1.6** | **1.9** |
|  | **0.9** | **1.1** | **1.7** |
|  | **0.6** | **0.9** | **1.3** |
|  | **0.7** | **1** | **1.5** |
|  | **1.1** | **1.6** | **1.9** |
| **Median  (Min , Max)** | **0.85**  **(0.6 , 1.1)** | **1.15 (0.9 , 1.6)** | **1.65 (1.3 , 1.9)** |

**Human C-peptide release in response to glucose challenge**

**MSCs co-cultured with uneducated exosomes**

|  | **Human C-peptide release (ng/μg protein/ hr)** | | |
| --- | --- | --- | --- |
|  | **5.5 mM** | **12 mM** | **25 mM** |
|  | **0.006** | **0.008** | **0.009** |
|  | **0.007** | **0.008** | **0.008** |
|  | **0.003** | **0.006** | **0.008** |
|  | **0.002** | **0.004** | **0.005** |
|  | **0.004** | **0.007** | **0.009** |
|  | **0.003** | **0.006** | **0.008** |
| **Median  (Min , Max)** | **0.0035**  **(0.002 , 0.007)** | **0.005 (0.004 , 0.008)** | **0.008 (0.005 , 0.009)** |


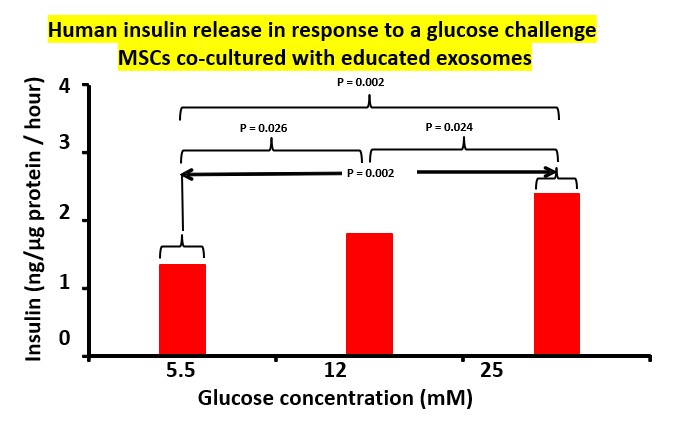
**Human insulin release in response to a glucose challenge
MSCs co-cultured with educated exosomes**

**Human C-peptide release in response to a glucose challenge
MSCs co-cultured with educated exosomes**


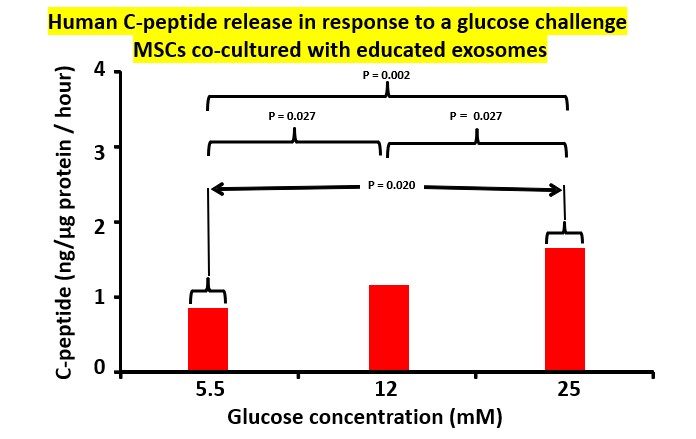


**Additional file 1: Data S12: uncropped gel of Western blotting**


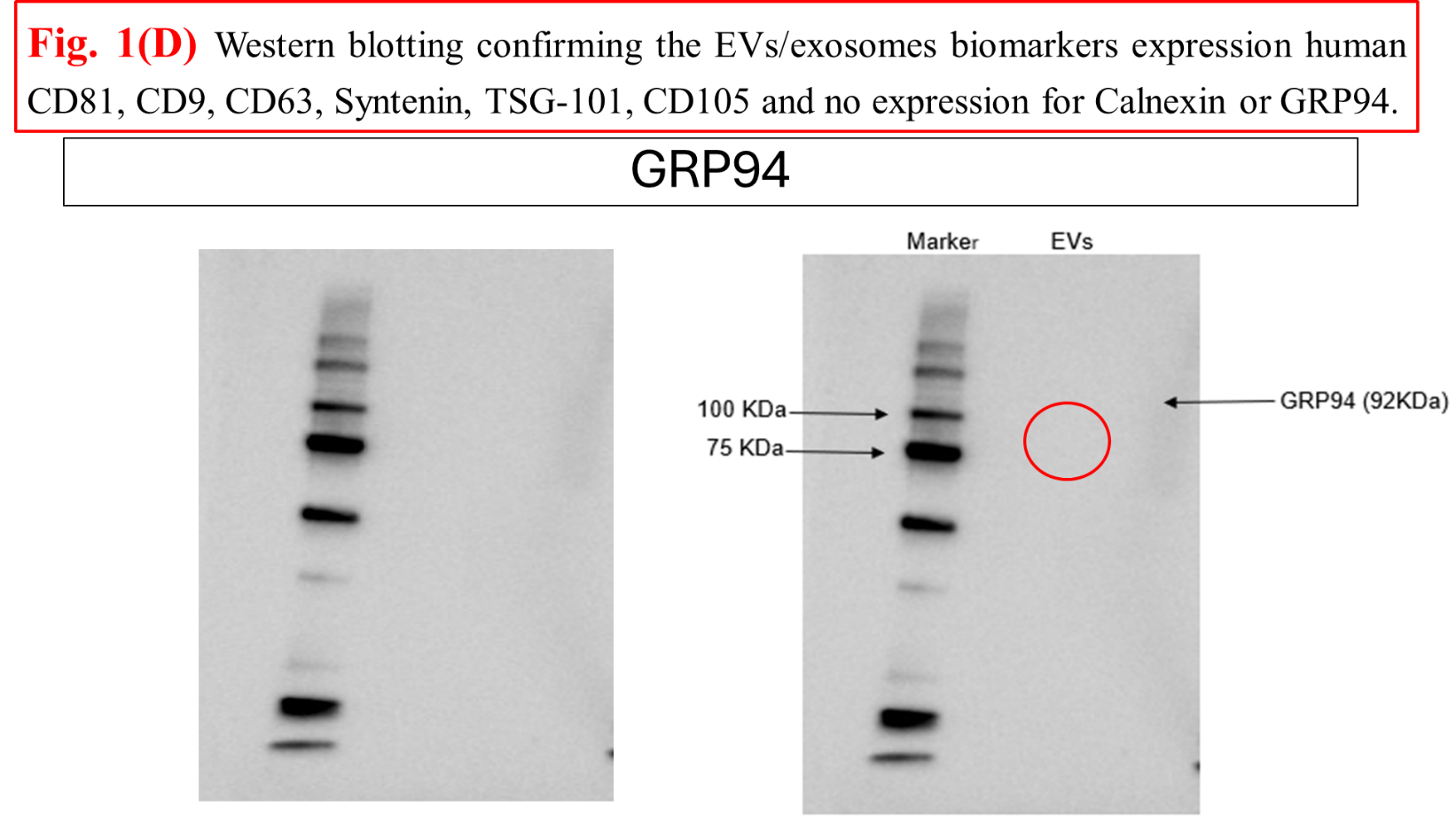


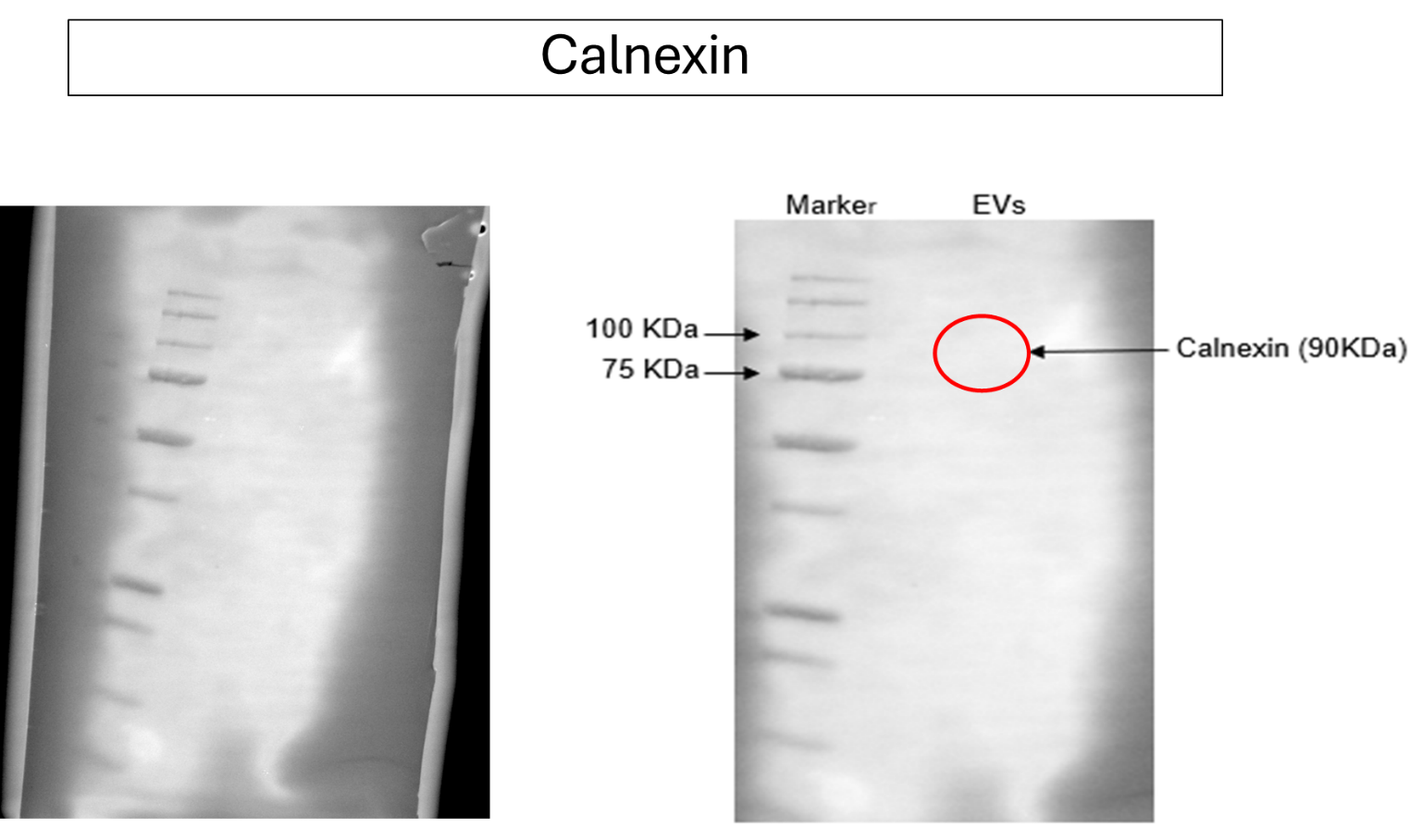


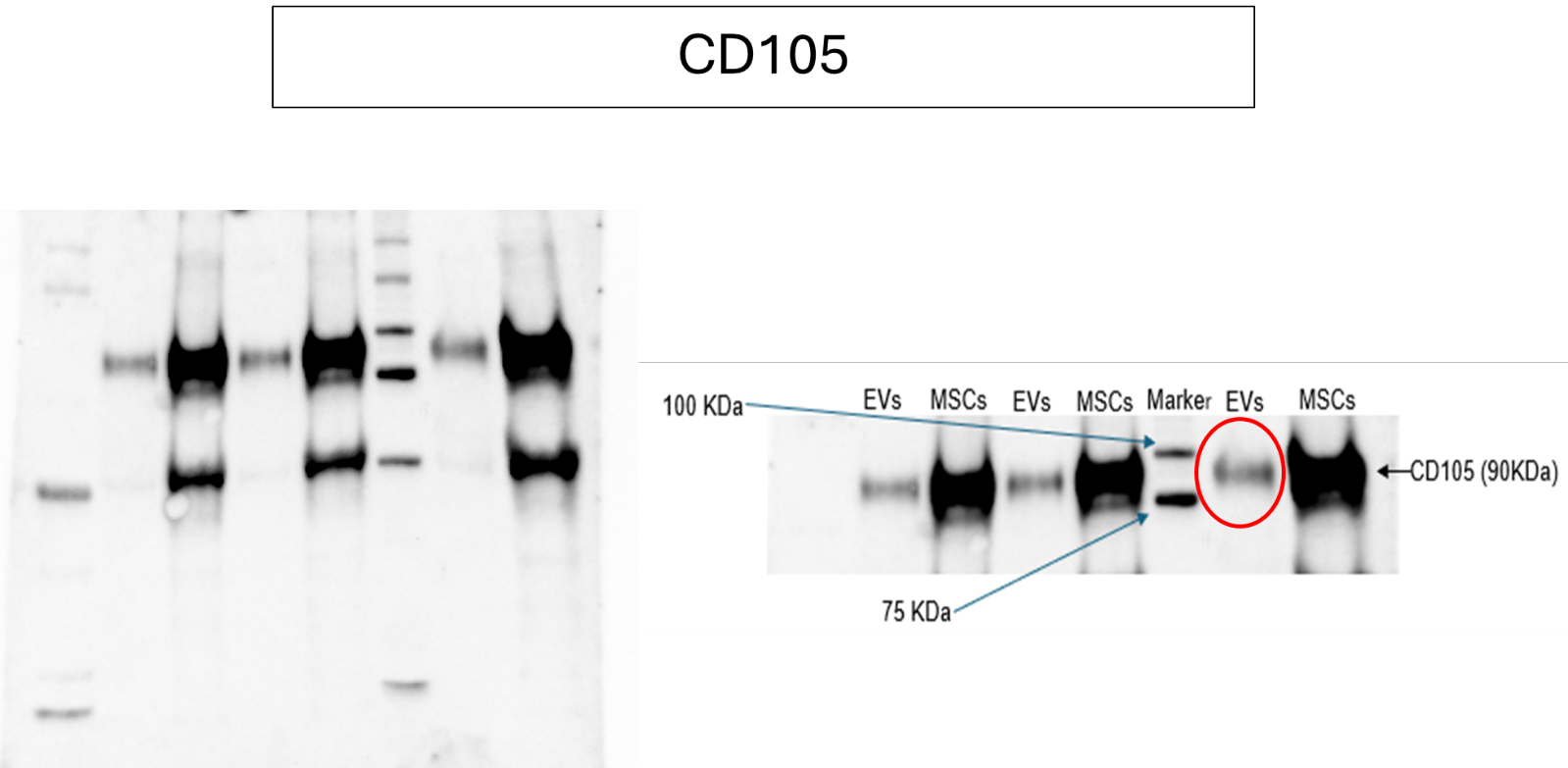


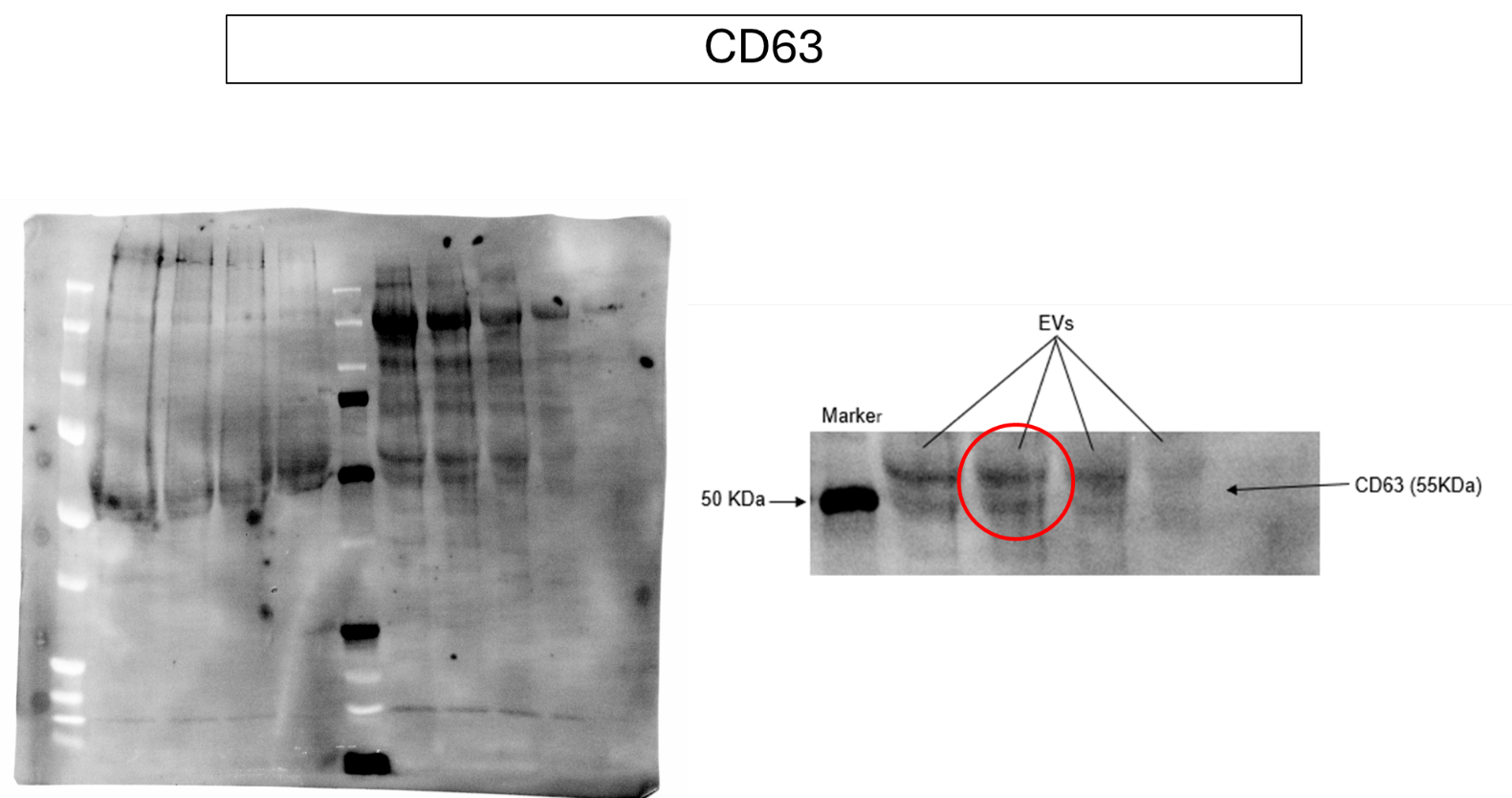


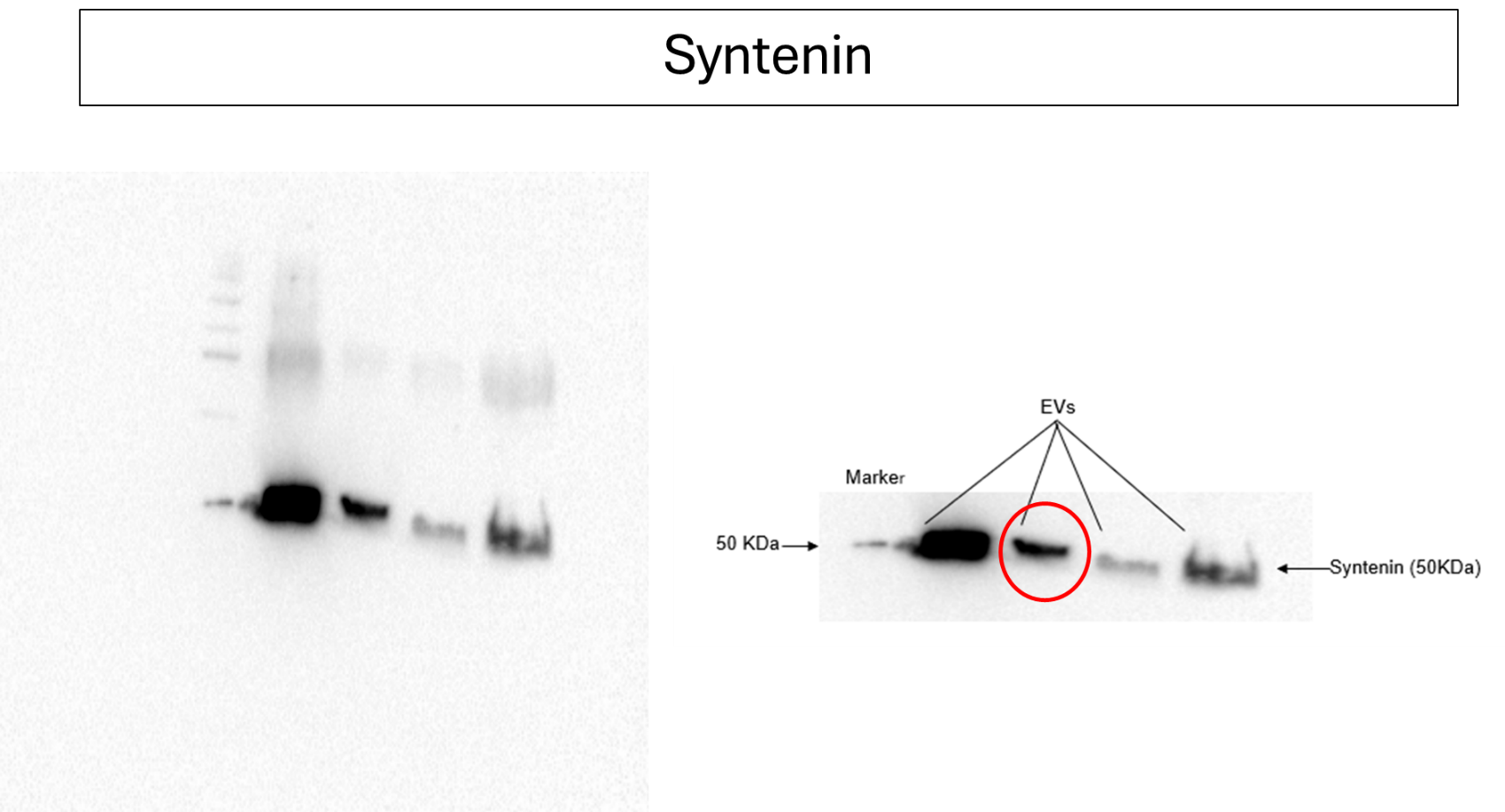


**
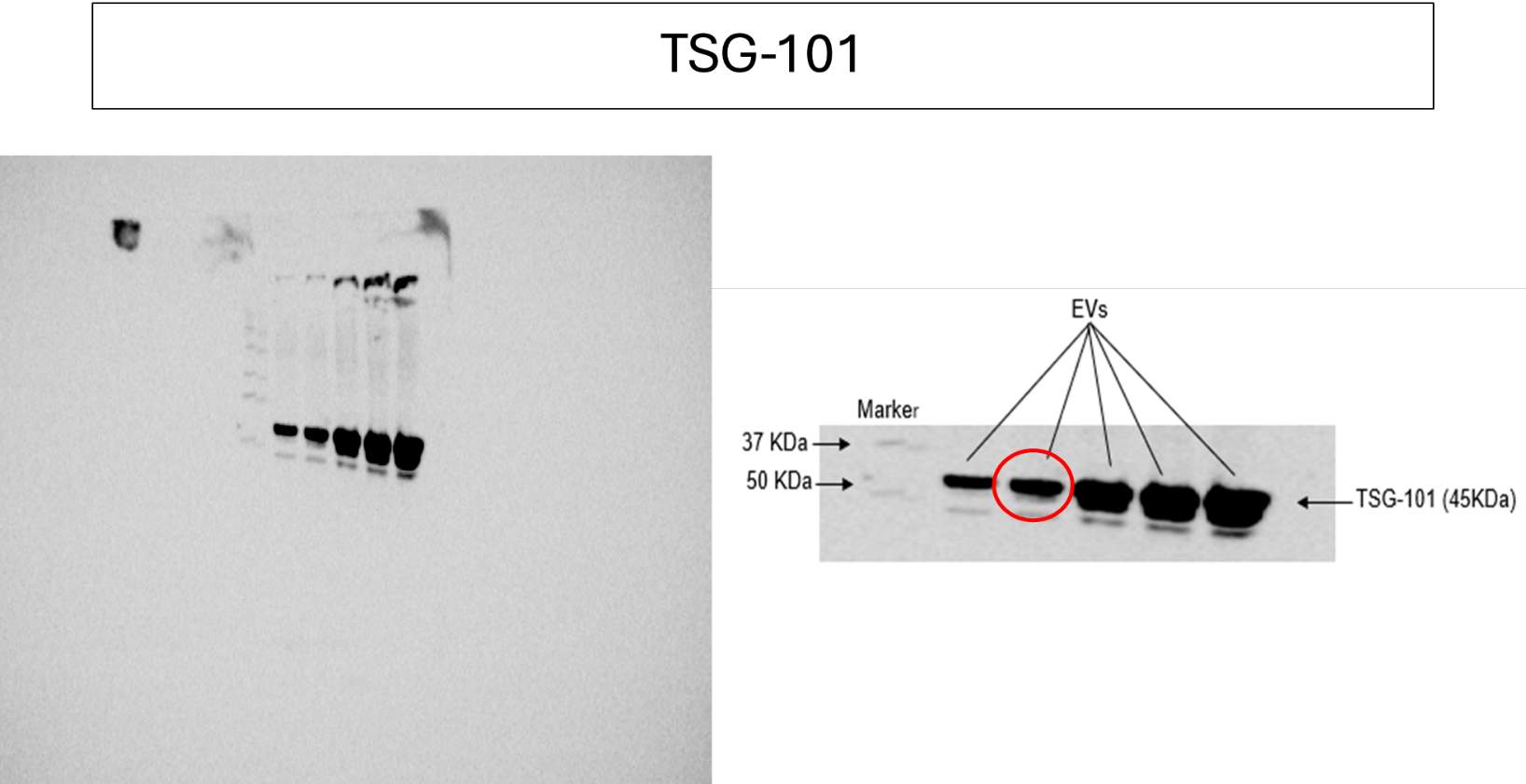
**


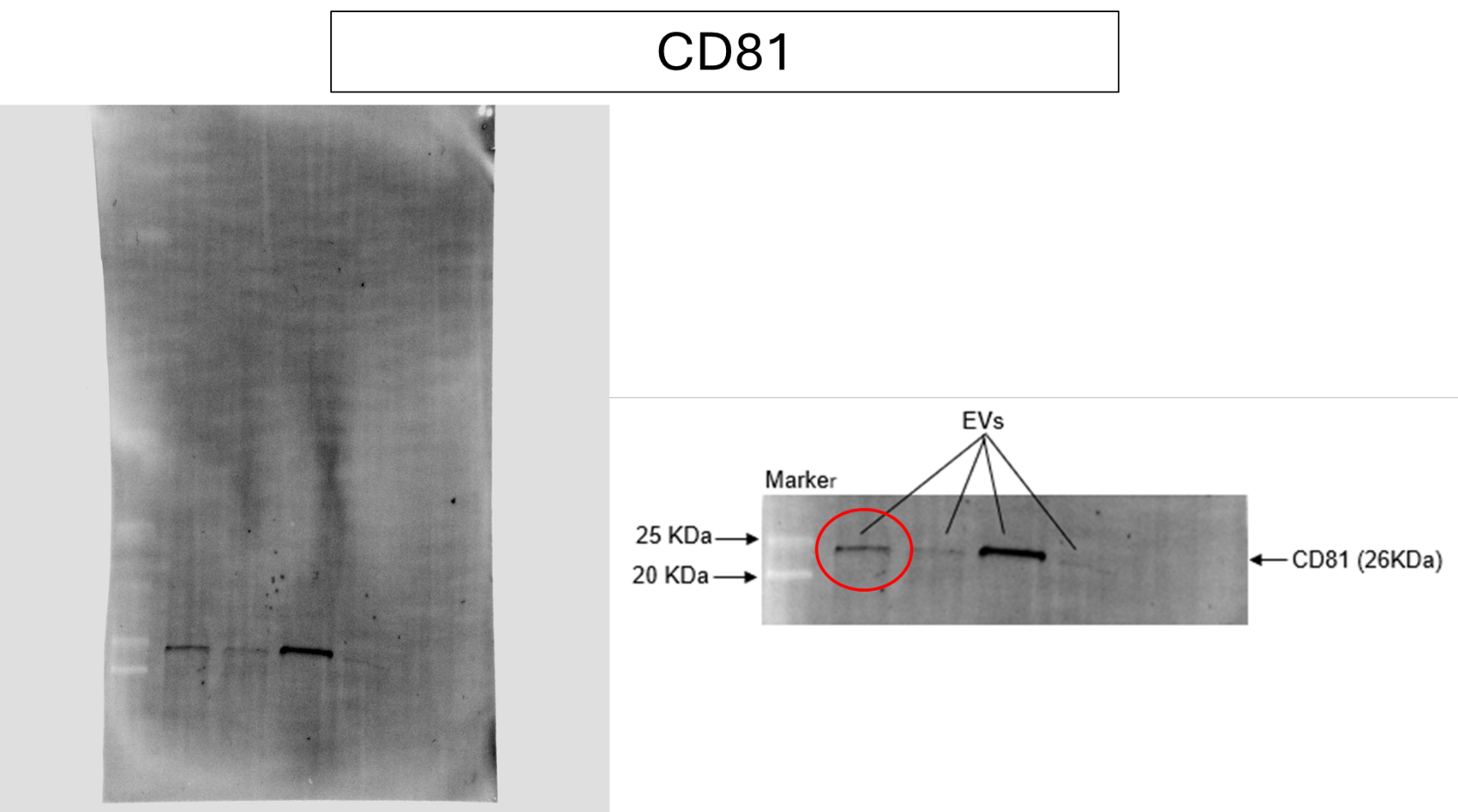


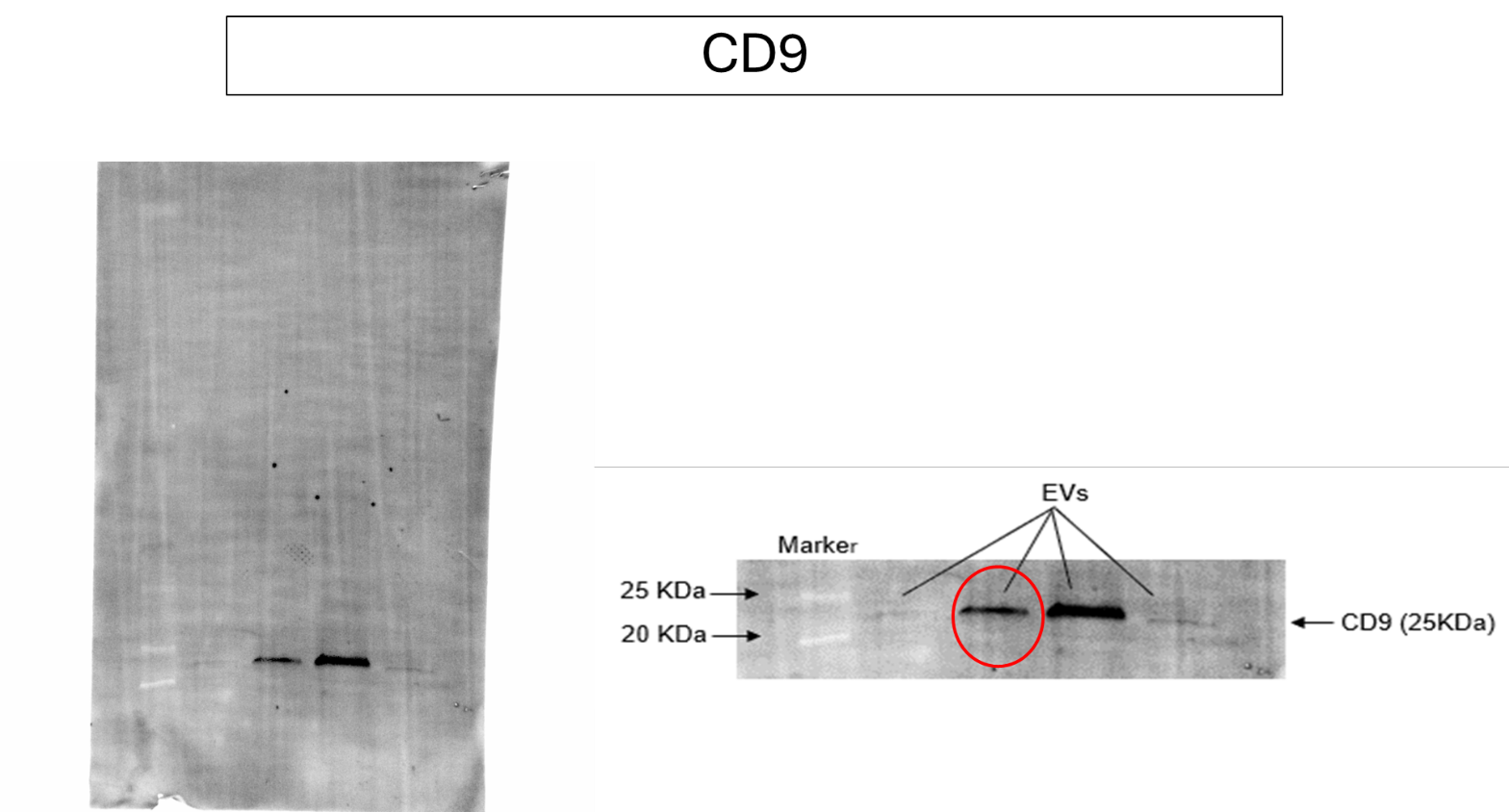

Supplement: Supplementary file 1 — Supplementary Information. [file 41598_2024_68104_MOESM1_ESM.docx]
